# Phage endolysins are adapted to specific hosts and are evolutionarily dynamic

**DOI:** 10.1371/journal.pbio.3001740

**Published:** 2022-08-01

**Authors:** Frank Oechslin, Xiaojun Zhu, Moira B. Dion, Rong Shi, Sylvain Moineau

**Affiliations:** 1 Département de biochimie, de microbiologie, et de bio-informatique, Faculté des sciences et de génie, Université Laval, Québec City, Canada; 2 Groupe de recherche en écologie buccale, Faculté de médecine dentaire, Université Laval, Québec City, Canada; 3 Félix d’Hérelle Reference Center for Bacterial Viruses, Université Laval, Québec City, Canada; Monash University, AUSTRALIA

## Abstract

Endolysins are produced by (bacterio)phages to rapidly degrade the bacterial cell wall and release new viral particles. Despite sharing a common function, endolysins present in phages that infect a specific bacterial species can be highly diverse and vary in types, number, and organization of their catalytic and cell wall binding domains. While much is now known about the biochemistry of phage endolysins, far less is known about the implication of their diversity on phage–host adaptation and evolution. Using CRISPR-Cas9 genome editing, we could genetically exchange a subset of different endolysin genes into distinct lactococcal phage genomes. Regardless of the type and biochemical properties of these endolysins, fitness costs associated to their genetic exchange were marginal if both recipient and donor phages were infecting the same bacterial strain, but gradually increased when taking place between phage that infect different strains or bacterial species. From an evolutionary perspective, we observed that endolysins could be naturally exchanged by homologous recombination between phages coinfecting a same bacterial strain. Furthermore, phage endolysins could adapt to their new phage/host environment by acquiring adaptative mutations. These observations highlight the remarkable ability of phage lytic systems to recombine and adapt and, therefore, explain their large diversity and mosaicism. It also indicates that evolution should be considered to act on functional modules rather than on bacteriophages themselves. Furthermore, the extensive degree of evolvability observed for phage endolysins offers new perspectives for their engineering as antimicrobial agents.

## Abstract 2

Endolysins are produced by bacteriophages to degrade the host cell wall and release new particles, but the implications of their diversity on phage-host adaptation and evolution is unknown. This study uses CRISPR-Cas9 genome editing to reveal novel insights into bacteriophage endolysin diversity and phage-bacteria interactions as well as into endolysin adaptation towards a new bacterial host.

## Introduction

Bacteriophages (phages) exhibit exceptional structural and genetic diversity [1]. A key feature of their genome organization is its mosaic gene composition, which results in the absence of universal genes. Still, individual genes or genetic regions can be shared between unrelated phage genomes [2]. Horizontal gene transfer between nonidentical ancestors is a major mediator of phage evolution, and phages that infect the same host may exhibit considerable diversity [3]. Phages are found in all studied biomes and are estimated to kill half of the global bacterial population every 48 h [4]. For this reason, phage-induced lysis is perhaps the most common fate for bacteria after cell division [5].

For most dsDNA phages, host lysis at the end of the replication cycle is due to the coordinated actions of 2 proteins. Holins are proteins that control the timing of lysis by permeabilizing the inner membrane of the host to allow the diffusion of the lytic enzymes, namely, the endolysins. The latter then gains access and degrades the cell wall peptidoglycan to induce lysis [5]. Destabilization of the outer membrane with the help of a third type of proteins called spanins is also required for phages that infect gram-negative bacteria [6]. In addition, some endolysins do not rely on holins but instead use signals to interact with the general host secretion pathway [7].

Peptidoglycan, the main component of the bacterial cell wall, provides mechanical resistance for cell integrity. It is composed of a complex meshwork of *N*-acetylglucosamine (GlcNAc)–*N*-acetylmuramic acid (MurNAc) glycan strands that are cross-linked by short stem peptides attached to MurNac residues [8]. Variation in the composition of the peptidoglycan has been observed between bacterial species, with approximately 100 types described to date [9]. Consequently, a wide variety of catalytic domains (CDs) has been observed among phage endolysins, which can cleave the glycosidic bonds between the sugar moieties (lysozymes/muramidases), the glycan–peptide linkage (amidases) and the stem peptide or its cross-bridge (endopeptidase) [10].

In addition to the CD, endolysins that target gram-positive bacteria usually have an additional C-terminal cell wall–binding domain (CBD) connected by a flexible linker [11]. Endolysins from phages that infect gram-negative bacteria rarely exhibit this modular organization and usually have only a CD [12]. CBDs are known to provide specificity for certain types of molecules present or associated with the peptidoglycan and can noncovalently attach to them [13]. Several CBDs have been described and include, among others, LysM domains. These domains interact with the sugar backbone of the peptidoglycan and are reported to be the most common [14,15]. The structure of these enzymes can also include more than 2 modules. Endolysins with 2 CDs and 1 CBD at the C-terminal [16,17] or central position [18,19] were reported from staphylococcal and streptococcal phages. Some endolysins can even be multimeric and composed of 2 gene products, as with the PlyC endolysin [20].

The endolysin diversity is illustrated in mycobacteriophages as 26 endolysin structures were observed through various combinations of 15 domains, even if the 220 analyzed phages were infecting the same *Mycobacterium smegmatis* mc^2^155 host [21]. One of the first metanalysis studies that described the genetic diversity of endolysins reported 89 types of structures from phages infecting 64 bacterial genera [12]. More recently, a database of 2,182 endolysin sequences was analyzed for possible correlations between domain families and bacterial hosts [22]. Remarkably, a clear differential distribution was observed between phages that infect gram-positive or gram-negative bacteria, except for amidase CDs, which were found in phages that infect both Gram types. In the case of gram-positive bacteria, amidase CD or LysM CBD were widely distributed. Conversely, other domains like PSA CBD or CPL1 CBD were restricted to phages infecting *Listeria* or streptococci. Importantly, no bacterial genus was associated with just 1 endolysin architectural composition.

The biological relevance for such diversity is not well understood. A possible explanation might be related to the evolutionary pressure that is put on phages to adapt their lysis system to a diversified and changeable bacterial cell wall [12]. It has also been proposed that the coevolution of phages along with their bacterial hosts selects for endolysin domains that target cell wall associated factors that are essential for host viability [23]. The presence of a specific type of endolysin could also be the result of an adaptation related to the phage’s preferred host. The PlyG endolysin only lyses specific *Bacillus anthracis* strains, which closely matches the host range of the phage [24]. Similarly, the CBD of *Listeria* phage endolysins Ply118 and Ply500 were shown to have a ligand binding specificity at the serovar level, like their respective phages [13]. Finally, a more provocative hypothesis would be that the observed endolysin diversity has no direct implication for the phage biology and is only a consequence of active domain exchanges between phages. Thus, one would expect that phage endolysins are interchangeable even between genetically unrelated phages.

In this study, we used phages that infect the gram-positive *Lactococcus lactis* as a model to study the roles of endolysin diversity on phage biology, host adaptation, and evolution. Analysis of 253 lactococcal phage genomes revealed 10 different types of endolysins, which were specific to phage groups. Although different from a biochemical perspective, genes coding for these endolysins and additional ones from phages infecting other bacterial species could be exchanged in different lactococcal phage genomes. The fitness costs were marginal if both recipient and donor phages were infecting the same bacterial strain, but gradually increased between phages that infect different strains or bacterial species. We also observed that phage lytic modules can be naturally exchanged between virulent phages and prophages. Finally, we showed that phage endolysins can rapidly adapt to their new phage/host environment by acquiring adaptative mutations.

## Results

### Endolysins of lactococcal phages can be grouped into 11 types

**Fig 1:**
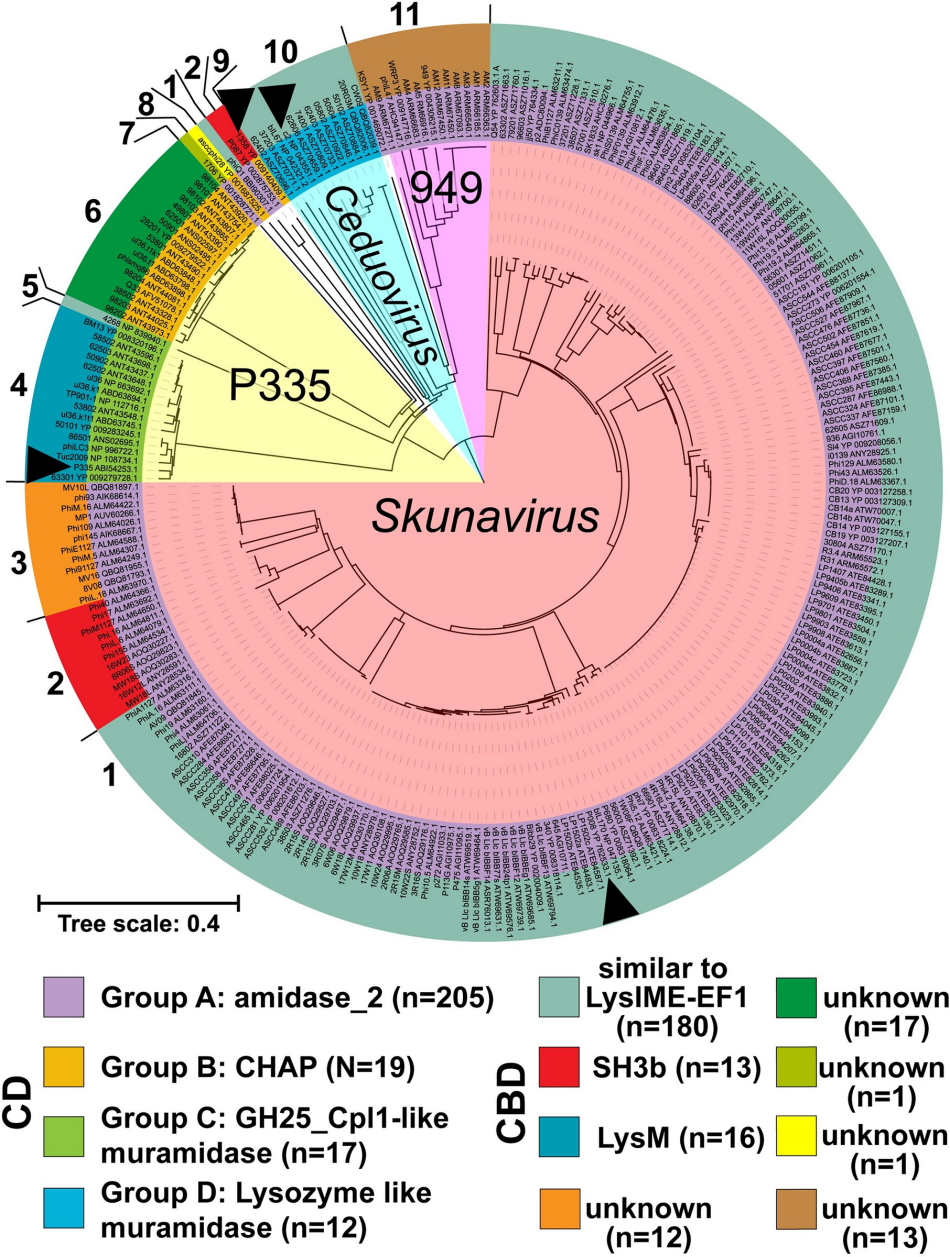
Phylogenetic relationship of endolysins of phages infecting *Lactococcus lactis*. The diversity of endolysins from 253 complete lactococcal phage genomes was investigated. We used ClustalW (v2.1) to perform multiple alignments and generate a phylogenetic tree (S1 Data). Conserved CDs and CBDs were determined based on HHPred and BLASTP predictions. The 253 endolysins were classified into 4 groups according to their CDs and are represented by different colors. Each phage is followed by the accession number of its respective endolysin. The phage taxonomic status is indicated when more than 1 species/genus is represented. CBD, cell wall–binding domain; CD, catalytic domain.

First, we investigated the diversity of endolysins in virulent and temperate phages that infect *L*. *lactis*. A set of 253 complete lactococcal phage genomes, ranging from 21,562 bp (phage 50504) to 132,949 bp (phage AM4) in length, were obtained from NCBI and analyzed for the presence of endolysins, conserved domains, and phylogenetic relatedness (Fig 1 and S1 Data). Four types of CDs (group A: amidase_2, group B: CHAP, group C: GH25_Cpl1-like, group D: lysozyme-like muramidase) were predicted based on HHPred and BLASTP.

Endolysins of group A have a deduced average molecular weight of 27.1 ± 2.8 kDa and are the most abundant as they were observed in 81% (*n =* 205) of these genomes. They all have a predicted N-terminal amidase_2 CD but are linked to diverse CBDs. Their C-terminal region shares homology with either the CBD of the *Enterococcus* phage endolysin LysIME-EF1 (phage 1706 and members of the *Skunavirus* genera) or the SH3b domain of lysostaphin (phage P087 and *Skunavirus* phages) or an unrecognized CBD (phages 1706, KSY1, and 949 as well as a few *Skunavirus*).

Endolysins of group B (7.5%, *n =* 19) have a molecular weight of 28.4 ± 3.1 kDa and a CHAP CD (Cysteine, Histidine-dependent Amidohydrolases/Peptidases). The cysteine and histidine residues, which are the hallmarks of CHAP domains [25], are conserved in all of them (cysteine at position 30 ± 3 amino acids and histidine at position 86 ± 5 aa; S1 Fig). Except for the endolysins from phage 1358, which had a SH3b-type CBD, the rest of the group B endolysins had C-terminal regions having no homology with known CBDs.

Endolysins of groups C and D were predicted to be muramidases. In group C (6.7%, *n =* 17), the GH25_Cpl1-like muramidase domain is associated with a predicted N-terminal transmembrane domain (TMD) of approximately 20 aa, followed by a signal peptidase I (SPaseI) cleavage site (CS) and 2 C-terminal LysM CBDs (46.3 ± 0.1 kDa) (S2 Fig). The only exception is the endolysin of phage 4268 (P335 group), which has a C-terminal sharing homology with the CBD of LysIME-EF1. In group D, the lysozyme-like muramidase CD (4.7%, 24.8 ± 0.7 kDa, *n =* 12) is always associated to a C-terminal region that shares homology with the CBD of LysIME-EF1.

Taken altogether, the combination of these CD and CBD domains resulted in 11 types of endolysins. In general, the phylogeny of the endolysins followed the taxonomy of lactococcal phages, including for the 3 most common groups (*Skunavirus*, *Ceduovirus*, and P335). Phages belonging to the *Skunavirus* genus have endolysins with amidase_2 CDs. Phages from the P335 group have endolysins with GH25_Cpl1-like and CHAP CDs, and *Ceduovirus* phages only had lysozyme-like muramidases.

As host cell lysis by lactococcal phages requires the presence of holins, we also investigated holin diversity and whether there is any correlation with the endolysin type. According to the number of TMDs (TMHMM tool), phylogenetic relatedness, and homology, holins were classified into at least 17 groups (S3 and S4 Figs and S7 Data). The group A endolysins found in *Skunavirus* phages was either associated with class II holins or class I holins with 3 putative TMDs. The other lactococcal phage groups that possessed group A endolysins either has class I holins (Q54), class II holins (P087), or class III holins with 1 TMD (KSY1, 1706, and 949). Group B endolysins were either associated with class II holins in P335 phages or type I holins in the 1358 and P034 groups. Endolysins from Group C were associated with class III holins or class II holins that belong to the phage_holin_1 and Dp1 superfamily. Group D muramidases were always associated with class I holins with 3 TMDs. Overall, we noticed that some types of holins were always associated with 1 type of endolysin, while other endolysins were associated with multiple types of holins.

### Endolysins from lactococcal phages are biochemically different

**Fig 2:**
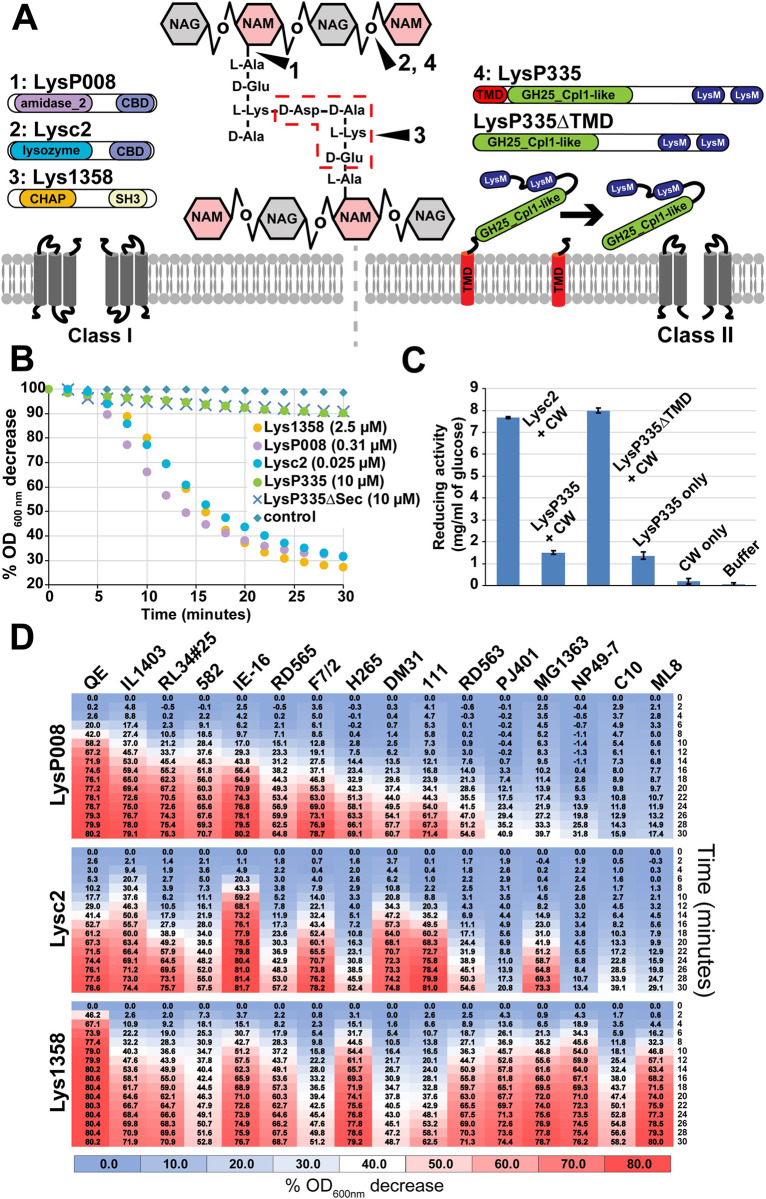
Biochemical characterization of the endolysins of the lactococcal phages P008, c2, 1358, and P335. (**A)** The endolysin of phage P008 (LysP008) is composed of an amidase_2 CD that is known to hydrolyse the glycan–peptide linkage and a C-terminal region with homology to the CBD of the endolysin found in the *Enterococcus* phage IMEEF1 [26]. The endolysin of phage c2 (Lysc2) has a lysozyme-like CD that can hydrolyse the glycosidic bonds between the sugar moieties and a C-terminal region that also shares homology with the CBD of phage IMEEF1 endolysin [26]. The endolysin of phage 1358 (Lys1358) has a CHAP CD and a predicted SH3 CBD. CHAP domains are endopeptidases that can hydrolyse the peptidoglycan crossbridge [25]. These 3 endolysins are associated with the class I holins and could be purified by affinity chromatography using a his-tag at the C-terminal of the enzyme. The endolysin from phage P335 (LysP335) was associated with a class II holin and has a more complex structure. LysP335 has an N-terminal predicted secretion signal composed of a TMD followed by a GH25_Cpl1-like domain with predicted glucosamidase activity and 2 C-terminal LysM CBDs. A predicted cleavage sequence was also observed after the TMD, which suggests that the LysP335 is exported to the periplasm in a holin-independent manner and stays at the membrane until it is released and activated through cleavage of its TMD. LysP335 and a construct without its TMD (LysP335ΔTMD) were also purified using affinity chromatography but with the addition of a TRX tag that was necessary for expression. (**B)** The lytic activity of the purified endolysins was characterized by following the decrease in turbidity of *L*. *lactis* IL1403 cells in exponential growth phase. The concentrations used corresponded to their specific activities, which were defined as the amount of enzyme needed to decrease the absorbance by 50% in 15 min (S1 Data). **(C)** As no lytic activity could be measured on *L*. *lactis* cells for the 2 purified LysP335 recombinant proteins, their enzymatic activity was tested on purified *L*. *lactis* IL1403 CW. Because LysP335 has a predicted glucosamidase activity, a Park–Johnson assay was used to measure glycan hydrolysis through the quantification of reducing groups, expressed in mg/ml of glucose equivalents released in the incubation mixture. The different endolysins (10 μM) were incubated with 100 mg/ml of cell wall. Solutions composed of the LysP335 endolysin, CW, or only buffer were used as controls (S1 Data). **D)** The host range of the endolysins LysP008, Lysc2, and Lys1358 was determined on several *L*. *lactis* strains. The activity of the endolysins was normalized for comparison and according to their specific activity measured on strain IL1403. The color gradient indicates the percent of decrease in absorbance measured over time. Values are means and standard deviations from triplicates (S1 Data). CBD, cell wall–binding domain; CD, catalytic domain; CW, cell wall; TMD, transmembrane domain.

We characterized 1 representative endolysin from each of the 4 CD groups. The genes coding for the endolysins of the virulent phages P008 (*Skunavirus*, LysP008, group A, amidase_2 CD and IMEEF1 CBD), 1358 (Lys1358, group B, CHAP endopeptidase CD and SH3b CBD), and c2 (*Ceduovirus*, Lysc2, group D, lysozyme-like CD and IMEEF1 CBD) were cloned into the expression vector pET28, introducing 6-His at the C-terminal position of the enzyme (Fig 2A). The endolysin gene from the virulent phage P335 (LysP335, group C, GH25_Cpl1-like muramidase CD and 2 LysM CBD) was cloned into the expression vector pETG20a to add a TRX-tag at the N-terminal position. This tag was necessary to improve the expression of the protein, which was also cloned both with and without its TMD (Fig 2A). The endolysins were overexpressed in *Escherichia coli* and purified using Ni-NTA affinity chromatography. The purity and molecular weight of each of the purified protein were verified on 4% to 12% BisTris gels (S5 Fig).

As previously mentioned, LysP008 and Lysc2 have a CBD with homology to the one present in the LysIME-EF1 endolysin [26]. Due to the presence of an alternative start codon before the CBD of LysIME-EF1, the endolysin was previously observed to form a tetramer composed of the full-length enzyme and 3 additional CBDs. We also observe that LysP008, Lysc2, and related endolysins appear to have an alternative start codon before their CBDs (S6 Fig). However, expression of the LysP008 and Lysc2 genes did not resulted in the production of 2 polypeptides (S6 Fig and S1 Data), even without codon optimization (S7 Fig). The enzymatic activity of the endolysins was tested by monitoring the turbidity decrease of a suspension of *L*. *lactis* IL1403 cells over time (Fig 2B and S1 Data). Our data clearly indicate that the addition of purified LysP008, Lys1358, and Lysc2 led to cell lysis, but LysP335 did not (with and without TMD). Although LysP335 could not lyse lactococcal cells, catalytic activity could still be measured when the enzyme was incubated with purified lactococcal cell walls (Fig 2C and S1 Data). Indeed, the hydrolysis of the glycan part of the peptidoglycan was observed with the LysP335ΔTMD construct only, and at a rate similar to Lysc2, which is consistent with their glucosamidase activity. The absence of activity with the full LysP335 suggest that the TMD must be removed to activate the enzyme. This might be achieved through the general secretion pathways and the action of signal peptidase I [27] as a SPaseI CS was observed between the TMD and the rest of the protein (S2 Fig). Moreover, LysP335 had to be purified as a membrane protein, which might indicate its location prior to its release and activation by proteolytic cleavage (Fig 2A).

LysP008, Lysc2, and Lys1358 also had different specific activities and host ranges. Lysc2 was the most active, as a concentration of 0.025 μM was enough to achieve a turbidity decrease of 50% in 15 min compared to 0.31 μM for LysP008 and 2.5 μM for Lys1358. To further compare their activity, each enzyme was normalized to a concentration needed to achieve a 50% turbidity decrease in 15 min using *L*. *lactis* IL1403. Next, these enzymes were tested on 18 *L*. *lactis* strains. Interestingly, variation in lytic activities was observed according to the type of endolysins and strains used (Fig 2D and S1 Data). Indeed, a turbidity decrease of at least 50% was observed after 30 min for 11 out of 18 (61%) tested strains for LysP008, 12 of 18 (67%) for Lysc2, and 16 of 18 (89%) for Lys1358.

### Phage P335 endolysin can be exchanged with one from another group without necessarily impacting phage growth

**Fig 3:**
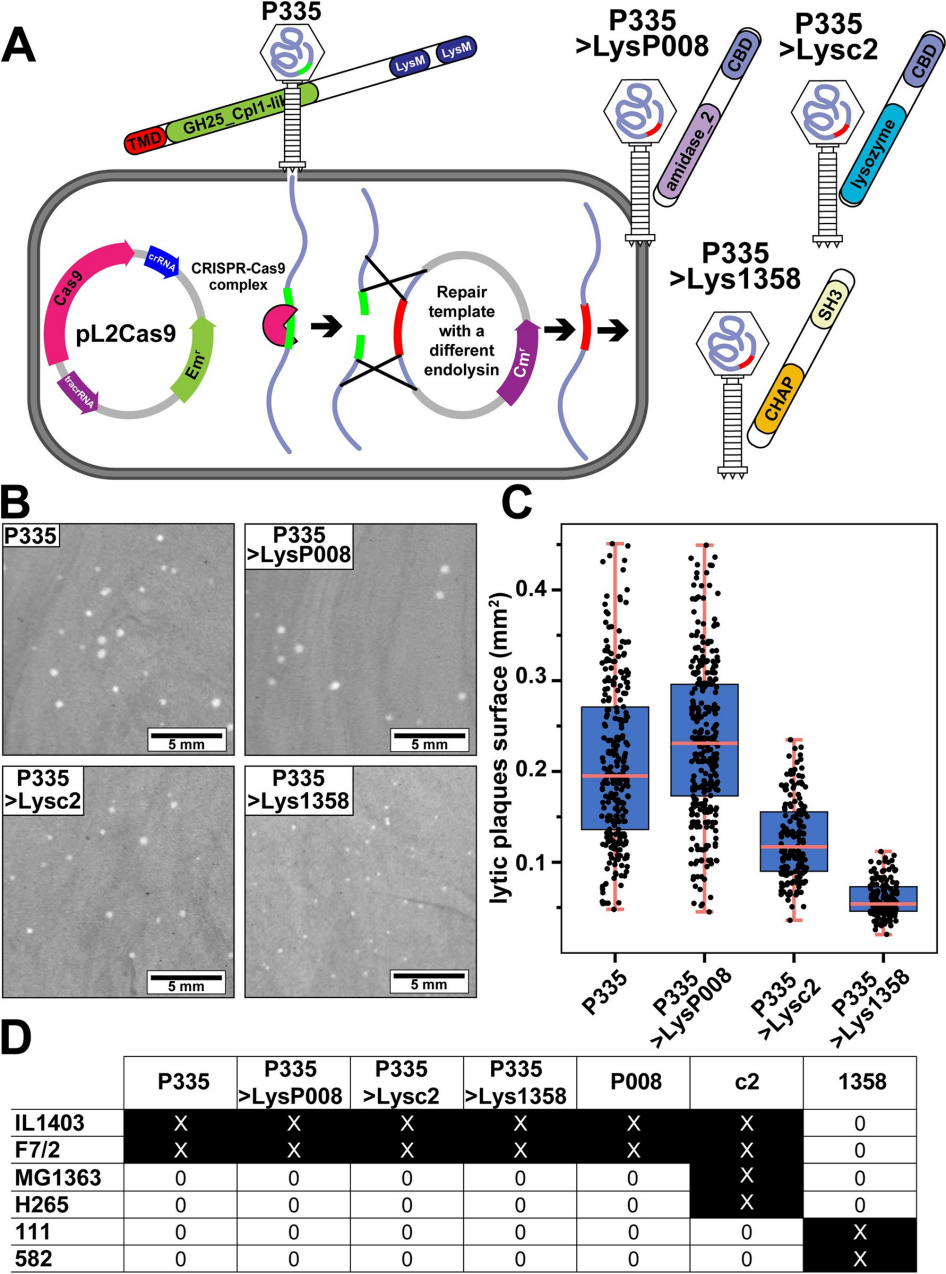
The gene coding for the endolysin in the virulent phage P335 was swapped by genome editing with homologs from other lactococcal phages. (**A)** Graphical representation of the genome editing of virulent phage P335 using CRISPR-Cas9. The gene coding for the endolysin of phage P335 was swapped with those from *L*. *lactis*-infecting phages P008, c2, and 1358. *L*. *lactis* strain IL1403 was transformed with 2 plasmids: (1) pL2Cas9-LysM1, to cleave the P335 genomic DNA within the endolysin gene; and (2) a second plasmid containing a repair template containing the gene to be exchanged. The endolysin gene in the repair template is flanked by 2 DNA fragments of ca. 250 bp of the LysP335 start and stop codons. The infection of this strain with phage P335 resulted in genomic cleavage within the endolysin gene, followed by a recombination event with the repair template that resulted in the endolysin gene exchange. After purification, phage P335 mutants with either the endolysin of phage P008 (P335>LysP008), c2 (P335>Lysc2), or 1358 (P335>Lys1358) were obtained. (**B)** Morphology and (**C)** size of the lytic plaques produced by phage P335 and the endolysin mutants (S1 Data). *L*. *lactis* IL1403 was used for plaque visualization using a double layer agar assay. (**D)** Host range of the phage mutants compared to the virulent wild-type phages. All measures were done in triplicates.

Despite sharing a common function, the endolysins of lactococcal phages are clearly phylogenetically and biochemically diverse. To study the implication of such a diversity in phage biology, we used the CRISPR-Cas9 genome editing tool to swap the gene coding for the endolysin of the virulent phage P335 with the endolysin-encoding gene from phages P008, c2, and 1358 (Fig 3A). The resulting virulent phage mutants, P335>LysP008, P335>Lysc2, and P335>Lys1358, were readily obtained and produced plaques (Fig 3B and 3C and S1 Data). While the plaque sizes were similar for phage P335 (0.21 ± 0.09 mm^2^) and the mutant P335>LysP008 (0.23 ± 0.08 mm^2^) (although statistically different, *p* < 0.001, Welch two-sample *t* test), the mutants P335>Lysc2 (0.12 ± 0.04 mm^2^) and P335>Lys1358 (0.06 ± 0.02 mm^2^) produced smaller plaques (*p* < 0.001, Welch two-sample *t* test). Of note, we did not observe any differences in the host ranges of the 3 phage mutants compared to the wild-type phages (Fig 3D).

**Fig 4:**
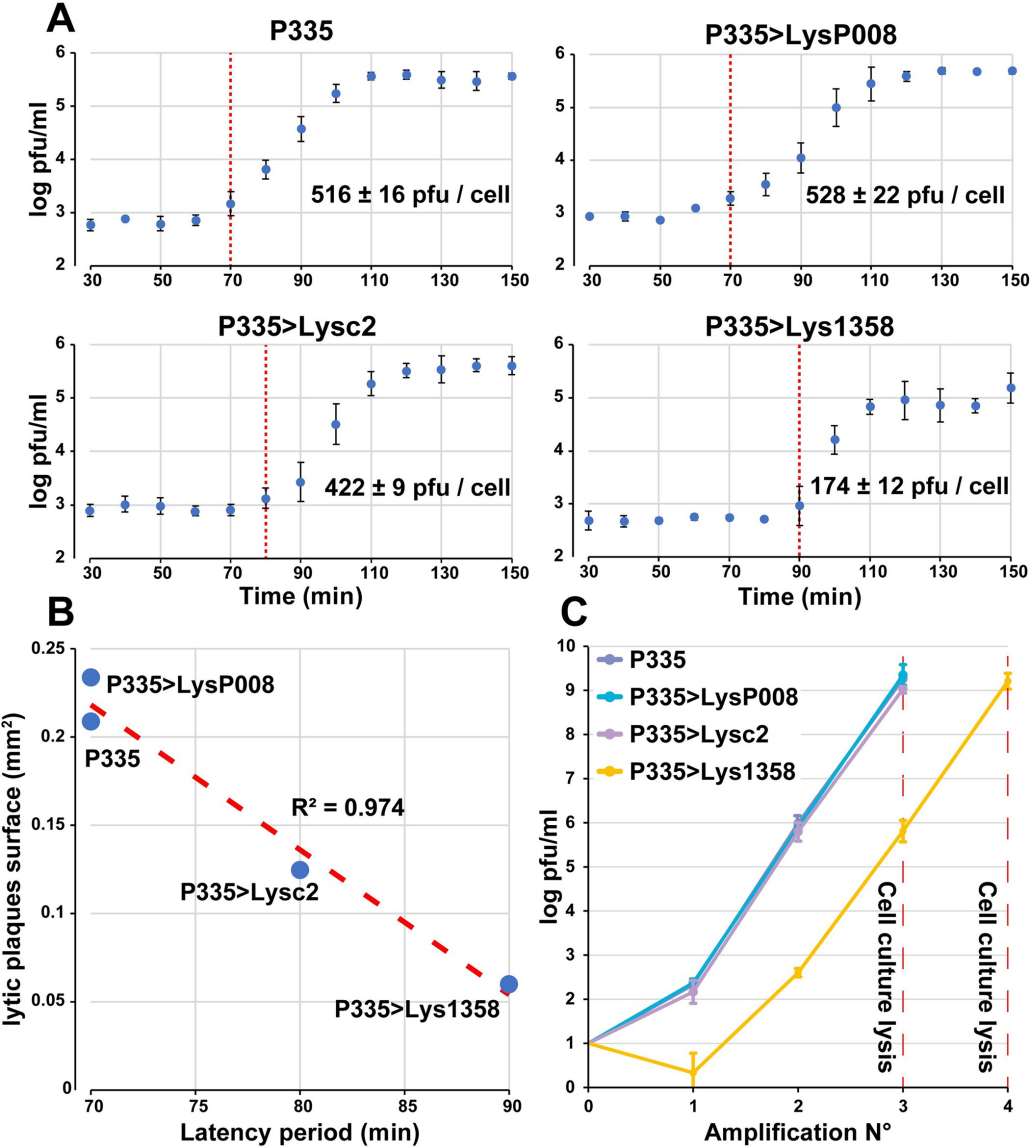
Characterization of the replication parameters of phage P335 and its endolysin mutants. **(A)** One-step growth curves of phage P335 and the endolysin mutants. The red dashed line indicates the end of the latency period (S1 Data). (**B)** Correlation between the mean size of the lytic plaques and the latency period of phage P335 and the endolysin mutants (S1 Data). (**C)** Replication dynamics of phage P335 and the endolysin phage mutants. *L*. *lactis* IL6288 cells in the exponential growth phase were infected with the different phages at a starting MOI of 0.000001. After the initial phage amplification that occurred during an overnight incubation, the bacteria and phages were transferred into fresh media at a dilution of 1/100 and amplified using the same conditions until clear lysis of the cell culture was observed. Phage titer was measured after each amplification step. Values are means and standard deviations from triplicates (S1 Data).

Phage growth parameters were further investigated using one-step growth curves. A latency period of 70 min was observed for phage P335, which ended 110 min after the start of infection and with an average burst size of 516 ± 16 new viral particles per infected cell (Fig 4A and S1 Data). Interestingly, exchanging the P335 endolysin with that from phage P008 (*Skunavirus*) did not have a significant impact, as similar latency period (70 min) and burst size (528 ± 22 PFU/cell) were observed. However, this was not the case for the P335>Lysc2 and P335>Lys1358 mutants, for which the latency periods were extended by 10 and 20 min, respectively (Fig 4A and S1 Data). Moreover, reduced burst sizes were observed for P335>Lysc2 (422 ± 9 PFU/cell) and P335>Lys1358 (174 ± 12 PFU/cell). We also observed a strong correlation (R^2^ = 0.974) between the plaque sizes and the latency period of the various phages (Fig 4B and S1 Data). The impact of the endolysin swap on phage fitness was also studied using a serial passage experiment (Fig 4C and S1 Data). Following phage titers over time, we observed that the mutants P335>LysP008 and P335>Lysc2 behaved similarly to P335 after 3 amplifications. This was not the case for P335>Lys1358, as we observed a reduction of 2 to 4 log/PFU during amplifications and it needed one more amplification to reach the same titer as the other phages.

### Endolysin genes can also be exchanged by homologous recombination between virulent phages and prophages

**Fig 5:**
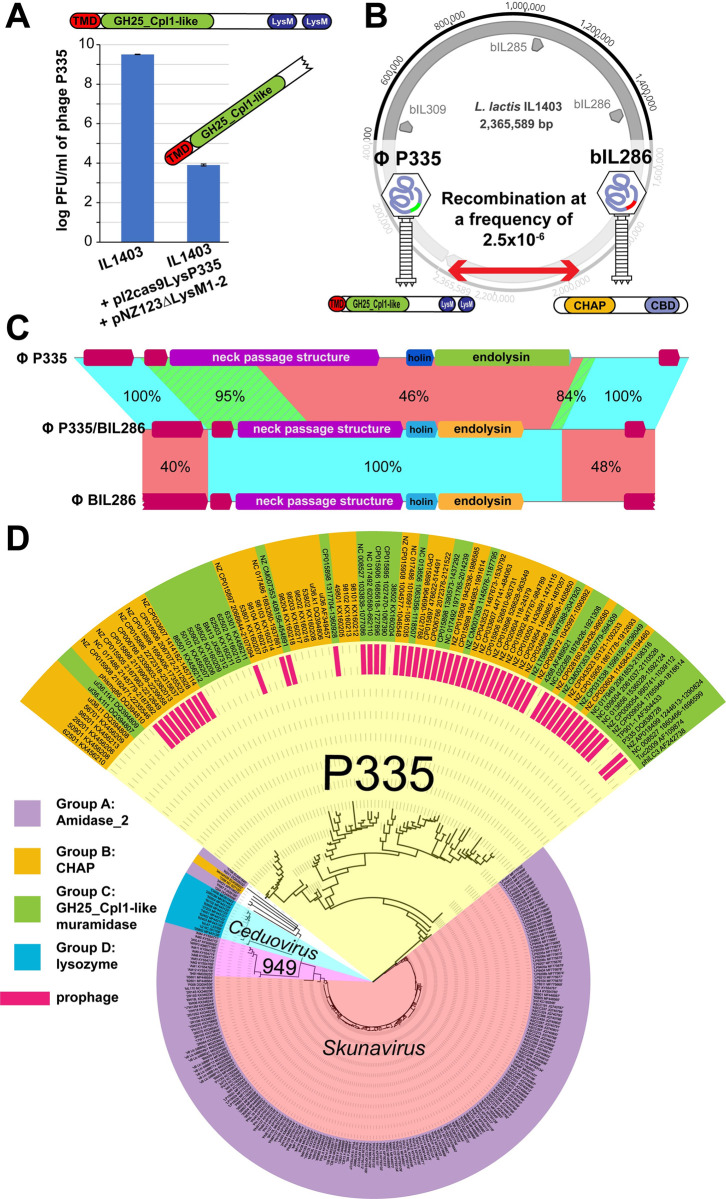
Exchange of endolysin-coding genes by homologous recombination between different phages. **(A)** Phage P335 was titered on the host strain *L*. *lactis* IL1403 and on the phage-resistant *L*. *lactis* IL1403 + pL2Cas9_LysP335_ + pNZ123_LysΔM1–2_ (S1 Data). **(B)** Graphical representation of the prophages integrated in the genome of *L*. *lactis* IL1403 and the recombination event that took place between the virulent phage P335 and prophage bIL286. (**C)** Graphical representation of the genomic region exchanged between P335 and bIL286 and includes part of the gene coding for the neck passage structure and the entire lysis cassette (holin and endolysin genes). (**D)** Phylogenetic analyses based on the genomes of lactococcal virulent phages and prophages (S1 Data). Sequence accession numbers are shown for virulent phages. For prophages, the positions of the start and end of the genomes are indicated after the accession number of the *L*. *lactis* accession number from which it was extracted. The type of endolysin CD and phage taxonomy is indicated by each color. CBD, cell wall–binding domain; CD, catalytic domain; TMD, transmembrane domain.

An interesting feature of the P335 endolysin (muramidase) was the presence of 2 LysM CBDs. We set up a CRISPR-Cas9 assay to remove these 2 domains and determine if they were essential for LysP335 activity. In situ deletion of the LysM domains was attempted using a strain carrying a Cas9-plasmid targeting the *lysP335* gene and a second plasmid (repair template) with *lysP335* without the sequence coding for the LysM domains. Infection of the recombinant strain with the virulent phage P335 resulted in an expected phage titer reduction (>5 logs, Fig 5A and S1 Data) due to Cas9 interference activity. Unexpectedly, we did not observe the anticipated deletion within the endolysin gene in the 16 recovered phages analyzed. Instead, these phages that had either a mutation in the protospacer adjacent motif (PAM) (4/16) or had acquired a different endolysin gene (12/16). Genome sequencing of 4 of these LysP335-negative mutants showed that they had acquired an endolysin gene from a known prophage (bIL286) of *L*. *lactis* IL1403. We estimated the recombination frequency at 2.5 × 10^−6^ (Fig 5B). Analyses of the recombinant phages (named P335/bIL286) indicated that a genomic region encompassing genes coding for a neck passage structure, holin, and type B endolysin was exchanged (Fig 5C). This exchange was likely due to homologous recombination, as the flanking genomic regions share homology between both the virulent phage P335 and the prophage bIL286 (Fig 5C). It also suggested that the deletion of the 2 LysM domains was detrimental, leading to the selection of these functional recombinant phages.

Due to this endolysin–gene swapping between virulent phages and prophages, we further investigated the genetic relatedness of our 253 phages and a set of 54 prophages retrieved from the genome of 26 *L*. *lactis* strains (Fig 5D and S1 Data). The analysis yielded results that were like the endolysin-based phylogeny, as phages were observed to cluster according to their endolysin types. However, the clustering was less obvious in prophages that belong to the P335 group, as some of them shared genetic similarities and were found to have either type B or C CDs. This was exemplified by the virulent phages ul36.k1t1 and ul36.t1 (86% coverage, 99.8% identity), phage 38502 and prophage NZ_CP015908_1004577–1046548 (46% coverage and 99.2% identity), or prophages NC_013656_1063356–1118507 and CP015898_964690–1021768 (54% coverage, 94.7% identity). The analysis further supported the possibility that different types of endolysins can be exchanged between related virulent phages as well as prophages.

### Endolysins from phages that infect *L*. *lactis* or other bacterial species can also be exchanged in the virulent phage P008

Having observed that endolysins with different architectures can be transferred in phage P335, we then addressed 2 other questions. First, we tried to determine whether it is possible to perform the same endolysin transfer in a different *L*. *lactis*-infecting phage and second, whether it would be possible to transfer endolysins from phages that infect other bacterial species. The virulent phage P008 was selected as it is different from P335 in many aspects: It has a type A endolysin, it is genetically unrelated, it has a much shorter latency period of 39 ± 1 min [28], and it produces larger plaques (6.34 ± 1.59 mm^2^).

**Fig 6:**
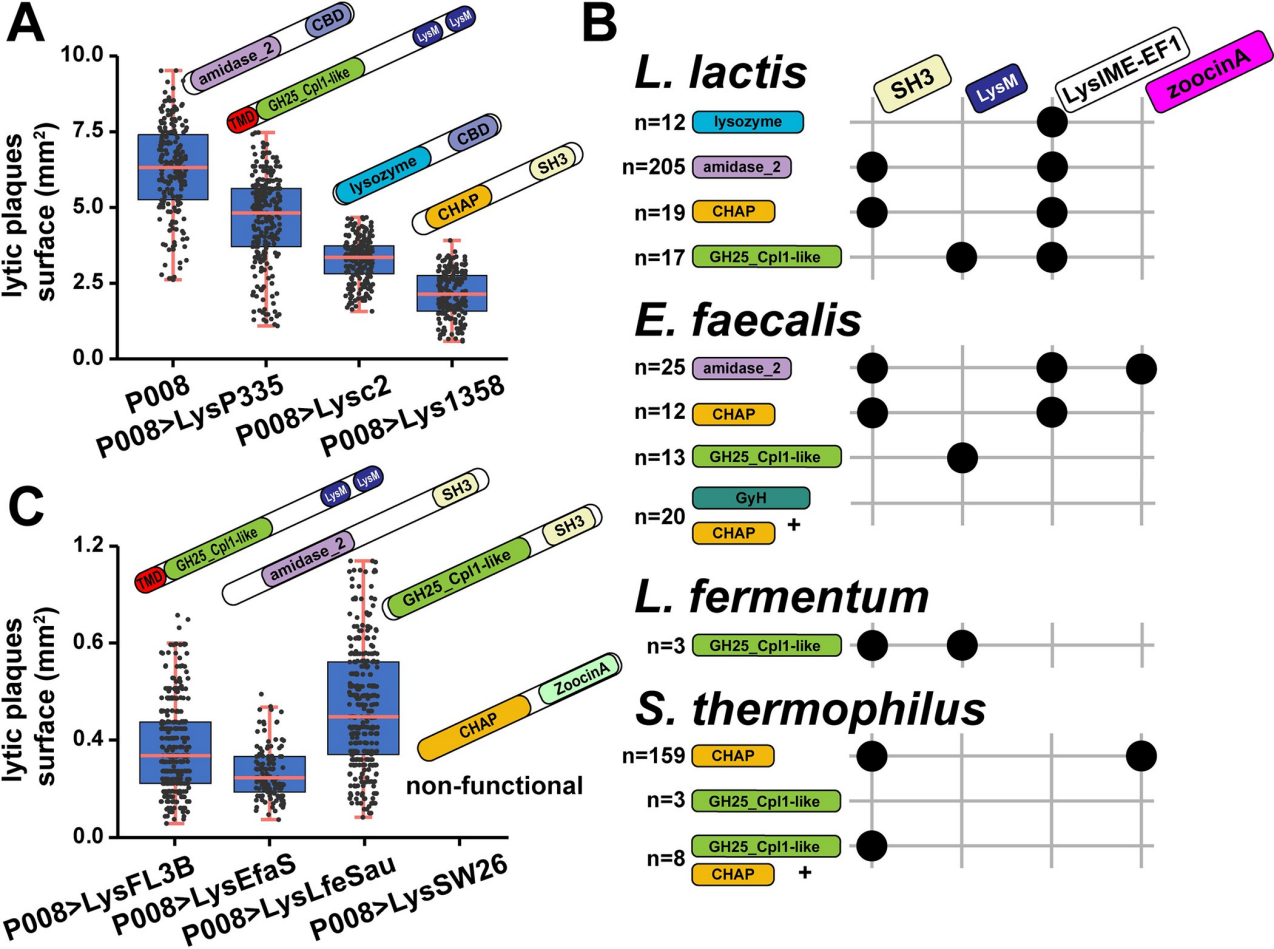
The gene coding for the endolysin in the virulent phage P008 was swapped by genome editing with homologs from other lactococcal phages or phages infecting other gram-positive bacteria. **(A)** Size of the lytic plaques produced by phage P008 and the mutants with an endolysin from phage P335 (>LysP335), c2 (>Lysc2), or 1358 (>Lys1358) (S1 Data). *L*. *lactis* IL1403 was used as host for plaque visualization using a double layer agar assay. (**B)** Endolysin diversity in phages infecting other gram-positive bacteria. Phages that infect *Enterococcus faecalis*, *Limosilactobacillus* fermentum, or *Streptococcus thermophilus* were selected as they share homology with lactococcal phages (S8 Fig and S7 Data). The association of a certain type of CD with CBD is indicated by a black dot. Graphical representation was inferred from the phylogenetic analysis of endolysin diversity in these phages (S9, S10, and S11 Figs and S7 Data). (**C)** Size of the lytic plaques of phage P008 and the mutants that contain the endolysins from the *E*. *faecalis* phages phiFL3B (ACZ64148.1, P008>LysFL3B) and vB_EfaS_Ef5.4 (QBZ69829.1, P008>LysEfaS), *L*. *fermentum* phage LfeSau (AIY32273.1, P008>LysLfeSau), and *S*. *thermophilus* phage SW26 (AYP29873.1, P008>LysSW26) (S1 Data). *L*. *lactis* IL1403 was used as the host strain. Measures were done in triplicates. CBD, cell wall–binding domain; CD, catalytic domain; TMD, transmembrane domain.

Transfer of the LysP335, Lysc2, and Lys1358 endolysins in P008 was straightforward and produced functional phage particles, as previously observed with phage P335. However, none of the new exchanged endolysins in these phage mutants led to plaque sizes that were comparable to phage P008 (*p* < 0.001, Welch two-sample *t* test) (Fig 6A and S1 Data). A trend similar to that of phage P335 endolysin mutants was observed, with phages P008>LysP335 producing the biggest plaques (4.60 ± 1.47 mm^2^), followed by phage P008>Lysc2 (3.27 ± 0.73 mm^2^) and P008>Lys1358 (2.12 ± 0.78 mm^2^) (S1 Data).

We then identified phages that infect other bacterial species but with either similar endolysins to lactococcal phages (at least 90% coverage and 50% identity) or homology at the genomic level (at least 1,000 bp homology). Our analyses indicated the presence of homology in phages that infect at least 12 bacterial species belonging to the *Streptococcaceae*, *Lactobacillaceae*, and *Enterococcaceae* (S8 Fig and S7 Data). Further analysis of endolysins in phages that infect strains of *E*. *faecalis* (S9 Fig), *Limosilactobacillus fermentum* (S10 Fig and S7 Data), and *Streptococcus thermophilus* (S11 Fig and S7 Data) revealed that they were primarily composed of CD and CBD domains comparable to the lactococcal ones (Fig 6B and S7 Data). Notable exceptions were the absence of type D–like endolysins, variation in the combination of the different domains, and the presence of zoocinA_TDR CBDs.

Endolysin genes from 4 phages that infect these different species were transferred into the genome of phage P008. These included the endolysins of the *E*. *faecalis* phage phiFL3B (LysFL3B, homolog of LysP335 with 59% identity and 100% coverage) and vB_EfaS_Ef5.4 (LysEfaS), *L*. *fermentum* phage LfeSau (LysLfeSau, GH25_Cpl1-like CD and SH3b CBD not observed in lactococcal phages), and *S*. *thermophilus* phage SW26 (LysSW26, zoocinA_TDR CBD not observed in lactococcal phages). Of note, phage P008 was favored here over P335 because it produces larger plaques, which facilitated the plaque observations. Except for LysSW26, the transfer of these new endolysins resulted again in functional phage particles, although the sizes of the phage plaques were much smaller than those observed with endolysins from lactococcal phages (*p* < 0.001, Welch two-sample *t* test) (Fig 6C and S1 Data). These data confirmed that endolysin genes can be swapped between lactococcal phages as well as with endolysins genes from phages that infect other bacterial species.

### Adaptation was observed during experimental evolution of the different phage endolysin mutants

**Fig 7:**
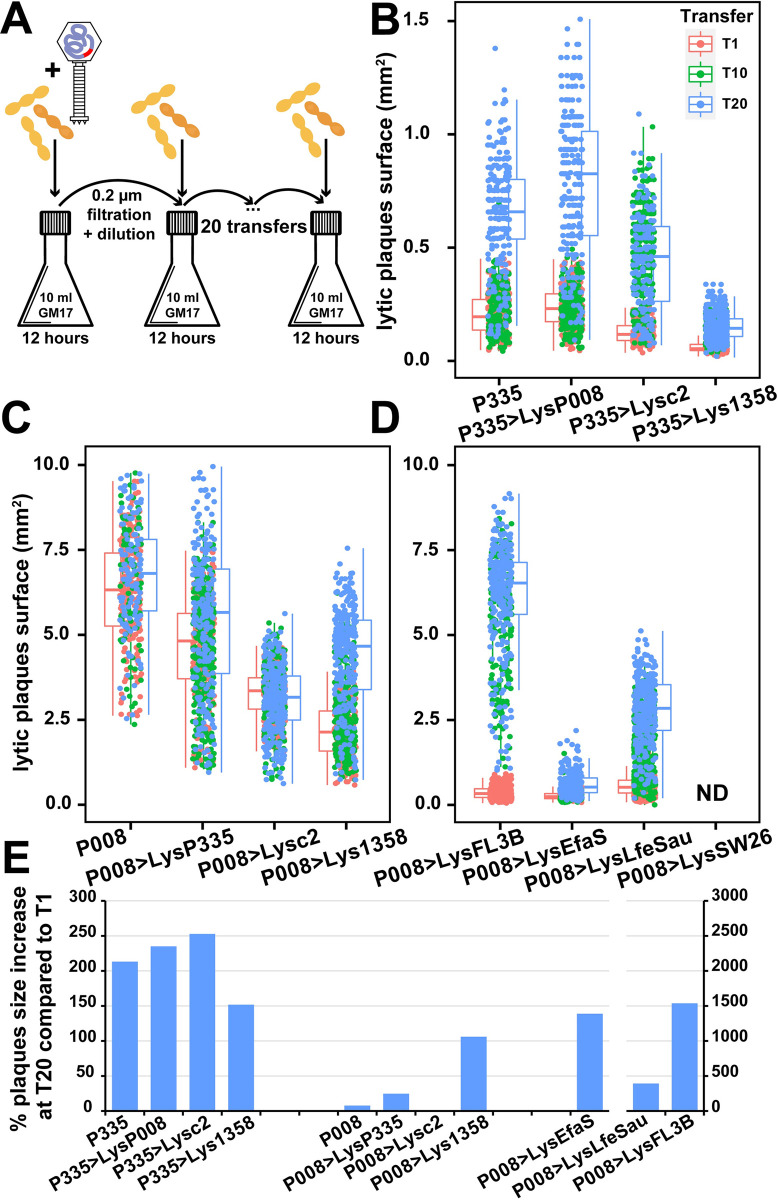
Experimental evolution of the phage mutants generated during the study. **(A)** Endolysin mutants from phages P335 and P008 were successively amplified for 20 transfers. *L*. *lactis* IL6288 cells in the exponential growth phase (OD_600nm_ 0.2) were infected at an initial MOI of 10. After an incubation period of 12 h at 30°C, phage lysates were filtered and diluted before another round of amplification was started. Dilutions of 1/1,000 were used for phages P335, P335>LysP008, P335>Lysc2, P335>Lys1358, P008>LysEfaS, P008>LysFL3B, and P008>LysLfeSau and dilutions of 1/10,000 for phages P008, P008>LysP335, P008>Lysc2, and P008>Lys1358. (**B, C, and D)** Size of the lytic plaques of the different phages after 1, 10, and 20 transfers (S1 Data). (**E)** plaque-size increased from T1 to T20 (S1 Data). Measures were done in triplicates.

As shown above, the exchange of endolysin gene may affect, in some cases, phage growth. Thus, we explored if these less efficient phage mutants would rapidly adapt to their new endolysins. We set up a short-term experimental evolution assay, in which the P335 and P008 phage mutants were amplified in 20 serial transfers (Fig 7A). Filtration of the infected cultures was performed between each of the transfers to ensure that only phages were allowed to carry over and evolve. First, we measured the plaque size of the phages after 1 (T1), 10 (T10), and 20 (T20) transfers to assess whether adaptation occurs during serial amplification. All phage mutants produced significantly larger plaques at T20 compared to T1 (*p* < 0.001, Welch two-sample *t* test), except for P008>Lysc2 (*p* = 0.0215) (Fig 7B–7D and S1 Data). When looking at the increase of plaque sizes from T1 to T20, larger differences were particularly observed among phage mutants with endolysins from phages infecting other bacterial species (Fig 7E and S1 Data). These data suggested that the phage mutants indeed adapted to their new endolysin gene, leading to an increase in their plaque size.

### Endolysins can adapt to their new phage/host environment

To investigate the genetic basis of adaptation to a new endolysin, the genome of the phages obtained after T10 and T20 were sequenced and compared to their wild-type counterparts. In the case of phage P335 and its endolysin mutants (P335>LysP008, P335>Lysc2, and P335>Lys1358), T20 sequencing indicated that they all recombined at the same position with another prophage (bIL285) found in the host of *L*. *lactis* IL1403. This resulted in the exchange of a large 18.5-kb genomic region that encompassed morphogenesis genes but not the bIL285 lytic system (S12 Fig). These recombination events appeared earlier (T10) for phage P335>Lysc2 and P335>Lys1358, as they were less fit than P335 and P335>LysP008 and likely provided a selective advantage.

**Fig 8:**
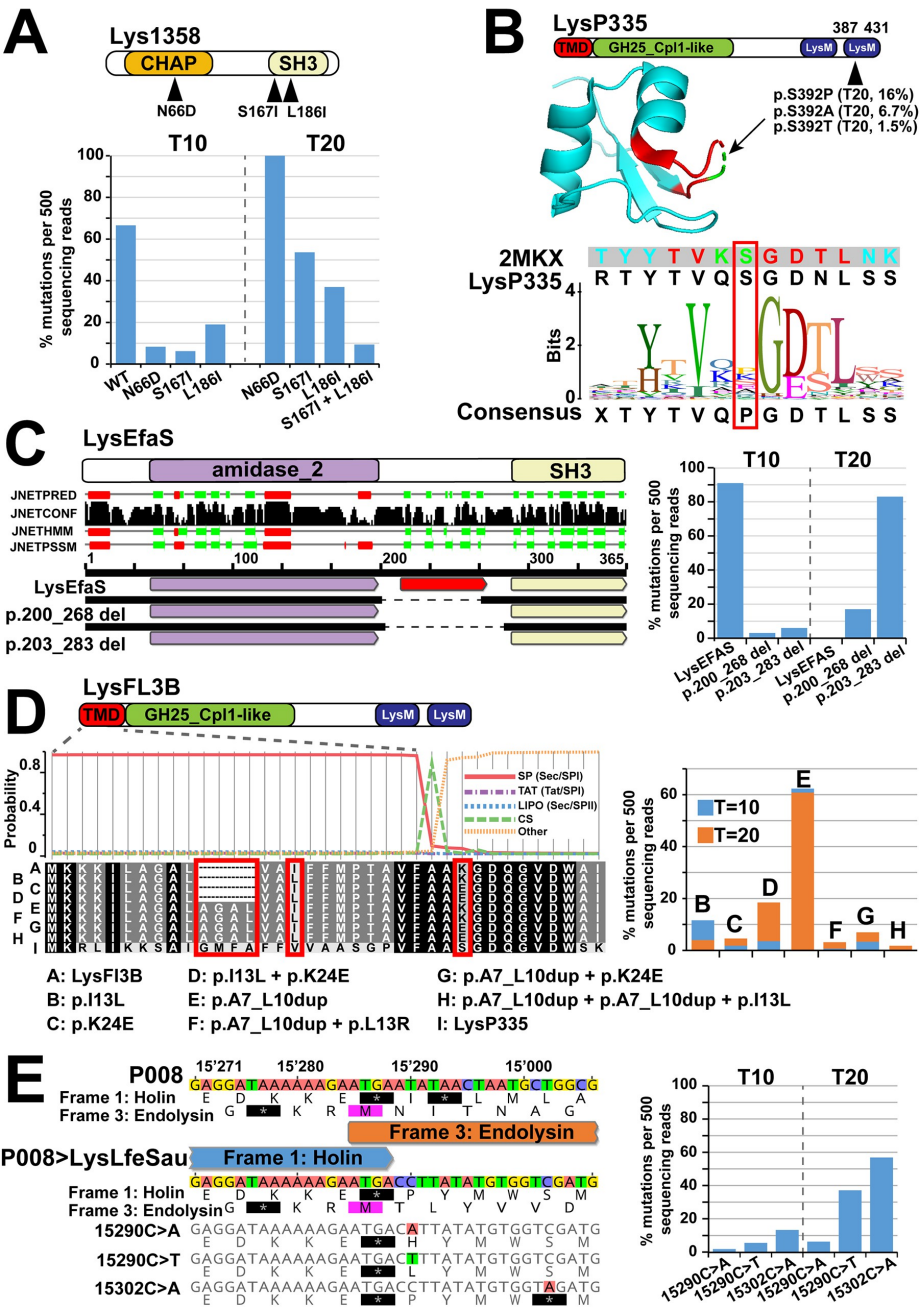
Genomic changes after experimental evolution of phage P008 with different endolysins after 10 and 20 transfers. (A) Three mutations (N66D, S167I, and L186I) were observed in the CHAP CD or SH3 CBD of the P008>Lys1358 mutant and appeared in different proportion in the T10 and T20 phage populations (S2 Data). (B) Phage P008>LysP335 had a mutation (S392P) in its LysM CBD, which was observed in ca. 16% of the phage population at T20 (S3 Data). The mutation was mapped, on the LysM domain of the *E*. *faecalis* autolysin AtlA, in a highly disordered loop and next to a glycine residue known to interact with the peptidoglycan stem peptide (15). (C) In the phage mutant P008>LysEFAS, the deletion of a domain composed essentially of beta strands and with no predicted function was observed. The deletion resulted in a different linker length between the amidase_2 and SH3 domains. The mutant with the shortest linker length was more present at T20 (S4 Data). (D) For the P008>LysFL3B mutant, duplication and missense mutations were identified in the N-terminal TMD and SPaseI CS of the enzyme (S5 Data). (E) For the P008>LysLfeSau mutant, mutations were found in the holin. In the phage P008, the end of the holin gene overlaps the start of the endolysin, with 2 imbedded stop codons. All mutants at T20 had missense mutations that introduce either a histidine or a leucine after the stop codon but did not result in amino acid changes in the frame of the endolysin or a second stop codon 12 bp later (S6 Data). CBD, cell wall–binding domain; CD, catalytic domain; CS, cleavage site; TMD, transmembrane domain.

The scenario was very different for the phage P008 mutants, as mutations were observed within the endolysins (Fig 8). For the phage P008>Lys1358 mutant, 3 mutations were present in the CHAP CD or SH3b CBD (N66D, S167I, and L186I; Fig 8A and S2 Data). For the CHAP domain, the N66D mutation was observed far away from the conserved residues C29 and H89 that are known to be essential for its catalytic activity [25]. Moreover, all mutations appeared at T10 in a similar low proportion. At T20, the N66D substitution in the CD appeared to be fixed in the population and was associated with either one of 2 mutations in the CBD but in a smaller proportion.

Similarly, the P008>LysP335 mutant had a mutation (S392P) in its LysM CBD (Fig 8B and S3 Data). An analysis of the sequencing reads indicated that the mutation appeared later and in 16% of the phages, which is consistent with Fig 7C. Importantly, the mutation was located next to a highly conserved region in all LysM domains. This region is conserved in the LysM domain of the *E*. *faecalis* autolysin AtlA for which a structure has been determined (PDB 2mkx). The S392 was mapped to a highly disordered part of a loop in the structure. Interestingly, the serine was mainly replaced by a proline, which is the most represented amino acid in the consensus sequence and can induce substantial conformational constrains. Moreover, the G393 residue located directly next to the mutation is known to interact with the peptide stem in the AtlA autolysin [15].

For the phage mutant P008>LysEFAS (Fig 8C and S4 Data), a deletion was observed in the linker region located between the amidase and SH3b domains of the enzyme. A secondary structure analysis of this region indicated the presence of a domain composed essentially of beta strands. In addition, the mutant with the shortest linker that connected the amidase_2 and SH3b domain (p.203_283 del) was more represented at T20, which suggests that the linker size may play an important role in enzyme optimization.

Two more adaptations were detected that were not related to the endolysin. For the P008>LysFL3B mutant, duplication and missense mutations were identified in the N-terminal part of the enzyme. The LysFL3B has an N-terminal TMD followed by an SPaseI CS that is most likely implicated in the regulation of the enzyme as observed for LysP335 (Fig 2D and S5 Data). Most of the mutants that appeared at T10 had a 4-amino acid duplication in their TMD, which became identical in length to the one observed in LysP335 and other phages that infect *L*. *lactis*. Additional less-represented mutations were also observed in this region or just after the type I Spase cleavage motif (A/V-X-A-A). In the P008>LysLfeSau phages, mutations were found to be related to the holin gene (Fig 2E and S6 Data). In the case of wild-type phage P008, the end of the holin gene overlaps the start of the endolysin, with 2 imbedded stop codons. A sequence analysis of the reads at T10 indicated the emergence of phage mutants with a missense mutation that introduces either a histidine or a leucine after the stop codon, but which did not result in amino acid changes in the frame of the endolysin or a second stop codon 12 bp later. These double mutants were more prevalent than the single mutant at T20.

Finally, additional mutations were detected in the rest of the wild-type phage P008 and mutant genomes. These mutations were in genes coding for the tape measure protein and the baseplate protein (S1 Table). These mutations were found in phages P008 or P008>Lysc2 for which no major plaque size increase was observed or were already present at T1 in the phage mutants. It is possible that these mutations have pleiotropic side effects, but they did not seem to provide a major fitness advantage in our laboratory conditions.

## Discussion

Endolysins are diverse, even in phages that infect the same bacterial species such as *L*. *lactis*. In lactococcal phages, 4 CD types were observed. These CDs were predicted to hydrolyze the peptidoglycan at 3 distinct positions and were also associated with several types of CBDs. The CD–CBD combinations resulted in the identification of 10 types of lactococcal phage endolysins. However, the biological relevance of such diversity and the implications on phage–host adaptation, fitness, and evolution is intriguing. To date, endolysins were mainly studied using phylogenetics, structural biology, and biochemistry-based methods that rarely consider phage biology. For example, the lytic activity of phage endolysins is often characterized using a turbidity decrease analysis of a bacterial cell solution after exogenous addition of the enzyme. While this approach is very useful from an application perspective of killing a targeted bacterium, it does not consider the physiological conditions in which these enzymes occur as they primarily function is from inside the cell.

Using a more holistic approach, we took advantage of CRISPR-Cas9 genome editing tool to study endolysins directly within a particular phage context. We generated functional phage mutants by transferring the genes coding for the endolysins of lactococcal phages P008, P335, c2, and 1358 into the genome of the virulent phages P335 and P008. From phylogenetic and biochemical perspectives, all 4 endolysins were associated with specific phage taxonomic groups and had different catalytic activities, mechanisms of regulation, and even host ranges. Moreover, the 2 engineered phages were genetically unrelated (belonging to different genera) and had different latency periods and holins. Yet, we were able to constructs these phage mutants with different endolysins. These results highlight the remarkable flexibility of the phage lytic systems and might explain the extensive diversity and mosaicism found in phage lytic modules [29]. Furthermore, these observations confirmed that the membrane pores produced by holins are nonspecific for the endolysins [30] and further suggest that endolysins biochemical parameters such as specific activity or host range are not sufficient to define endolysin contribution to phage biology.

While the exchange of endolysin genes was obtained using a genome editing tool, we did also notice natural recombination events with phage P335. Indeed, the virulent phage P335 could naturally acquire a different endolysin-coding gene that was present in a prophage through homologous recombination, as both viral genomes shared regions of homology. DNA exchange between a P335-like phage and its host chromosome was previously reported at a similar frequency and in response to a phage defense mechanism (abortive infection), although these transfers did not involve endolysins [31]. It is tempting to speculate that bacterial defense systems that specifically target the phage lytic system may also provide a selective pressure to favor endolysin exchanges.

In mycobacteriophages, intragenic mosaicism mediated by illegitimate recombination between functional domains was also proposed as a driver for the extensive endolysin diversity [4]. We did not observe intramolecular mosaicism in endolysins from phages that infect *L*. *lactis*. Specific CDs were rarely associated with more than 1 specific CBD and were shared between different lactococcal phage groups. The endolysin diversity might be the result of past genetic exchanges with phages infecting other bacterial species that resulted in the introduction of new endolysin types in lactococcal phages. It was previously suggested that some phages that infect *L*. *lactis* possibly recombined with or even emerged from phages that infect strains from the phylum Firmicutes [32–35]. To explore this possibility, we introduced endolysins from phages that infect *E*. *faecalis*, *L*. *fermentum*, and *S*. *thermophilus* strains into lactococcal phages. With the exception of the endolysin from *S*. *thermophilus* phage SW26, the swapping of endolysins with either structural homology or new interdomain associations could produce functional lactococcal phage particles. The success of these endolysin exchanges is likely related to the peptidoglycan composition of the different host species from which the viral endolysins were isolated. For example, the inability to transfer the LysSW26 endolysin may be explained by the specificity of its CHAP CD and ZoocinA_TDR CBD toward peptidoglycan cross-bridges [25,36], which are not conserved between *L*. *lactis* (L-Lys-D-Asp) and *S*. *thermophilus* (L-Ala_2_ or, alternatively, L-Ala_3_) [9]. On the other hand, the endolysin types that produced functional lactococcal phage recombinants had domains that target more conserved structures. The amid bond that is targeted by the amidase_2 domain of LysEfas or the glycan fraction (GlcNAc-MurNAc) that is hydrolyzed by the GH25_Cpl1-like domain of LysFL3B are conserved between the bacterial species. For the same reason, the GH25_Cpl1-like domain of LysLfeSau was functional, even if *L*. *fermentum* cells have L-ornithine instead of L-Lys in the stem peptide (L-Ala-g-D-Glu-L-Orn-D-Ala) and D-Asn-D-Ala for the cross-bridge. These results also confirm that “generalist” endolysins with catalytic activities targeting conserved peptidoglycan features are more prone to being transferred between phages that infect different bacterial genera [22].

Still, fitness costs were noted for most of the recombinant phages and were always smaller when the endolysins were swapped between phages that infect the same strain as compared to between phages infecting different strains or species. Interestingly, recombinant phages with such fitness costs were found to readily adapt in a relatively short time. Using an experimental evolution assay, we observed that adaptation can specifically take place in endolysins genes by following a very wide range of mutations. Adaptation could arise by acquisition of point mutations in the CD or CBD and at positions that were sometimes close to amino acids that interact with the substrate. Furthermore, deletion of entire domains, variation in the length of the linker connecting them, and modification of parts involved in the regulation of the enzyme was also observed. These results indicate that endolysins have high degree of evolvability and likely explain their fixation in a given phage group.

In conclusion, our observations highlight the remarkable ability of phage lytic systems to recombine and adapt and therefore explain their large diversity and mosaicism. It also indicates that evolution should be considered to act on functional modules rather than on phages themselves. From an application perspective, the availability of the CRISPR-Cas technology and the extensive degree of evolvability observed for phage endolysins offers new perspectives for their engineering as antimicrobial agents. Indeed, the phage replication machinery could be used to generate adaptation and increase the antimicrobial activity of the different endolysins toward a specific pathogenic bacterium. Furthermore, the adaptation process could also generate mutational fitness landscapes that highlight important residues for the activity and specificity of these enzymes, which will be an essential step for any future engineering efforts.

## Methods

### Bacterial strains, phages, and growth conditions

All bacterial strains, phages, and plasmids used in this study are listed in S2 Table. *L*. *lactis* strains and phages P335 and P008 were obtained from the Félix d’Hérelle Reference Center for Bacterial Viruses (www.phage.ulaval.ca). *L*. *lactis* strains were grown statically at 30°C using M17 broth with 0.5% glucose (GM17) and 1.0% (w/v) agar for solid media. When needed, chloramphenicol or erythromycin was added to the media at a final concentration of 5 μg/mL (Cm 5 or Em 5). *E*. *coli* strains were cultured at 37°C in LB with agitation (220 rpm) or plated on LB with agar (LBA). When necessary, LB was supplemented with kanamycin sulfate (30 μg/mL), chloramphenicol (25 μg/mL for LBA plates and 50 μg/mL for LB), or ampicillin (100 μg/mL). For phage infection, 10 mM CaCl_2_ was added to the media and double layer plaque assays were performed as previously described [37].

### Plasmid construction

Plasmids were purified from overnight bacterial cultures using a QIAprep Spin Miniprep kit (Qiagen). Lysozyme was added to a final concentration of 30 mg/ml for *L*. *lactis* cultures. Polymerase chain reactions (PCRs) were performed with Taq polymerase (Feldan) for screening purposes or Q5 high-fidelity DNA polymerase (New England Biolabs) for cloning. A Gibson assembly master mixture was prepared as described previously [38]. Restriction enzymes were purchased from New England Biolabs. Primers used in this study are listed in S1 Table.

### Phylogenetic analysis of the endolysin and holin amino acid sequences of virulent or temperate lactococcal phages

We queried 253 genomes from phages that infect *Lactococcus* for their holin and endolysin genes and deduced amino acid sequences. We used ClustalW (v2.1) to perform multiple alignments and to generate phylogenetic trees for both holin and endolysin amino acid sequences, using the default parameters [39]. Phylogenetic trees were visualized and rerooted using Interactive Tree Of Life (iTOL) v5 [40]. The conserved domains of both endolysins and holins were determined according to HHPred and BLASTP predictions [41,42]. TMD predictions were performed using the TMHMM tool [43]. Signal peptides and CSs were identified using the SignalP 5.0 server [44].

### Cloning, expression, and purification of the endolysins from phages P335, P008, c2, and 1358

The genes that code for the endolysins LysP335 (ABI54253.1), LysP008 (AAY97824.1), Lysc2 (NP_043551.1), and Lys1358 (YP_009140409.1) were synthetized with an additional codon optimization for *E*. *coli* BL21 (Integrated DNA Technologies). Complementary ends (35 bp) were added at the 5′ and 3′-end of an NcoI/XhoI linearized pET28a to allow cloning of the genes by Gibson assembly. Subsequent plasmids named pLysP335^28a^, pLysP008^28a^, pLysc2^28a^, and pLys1358^28a^ were transformed in One Shot BL21(DE3) chemically competent *E*. *coli* cells (Life Technologies) and propagated on LB plates or broth supplemented with kanamycin. Plasmid pLysP335^28a^ was also used as a template to subclone either LysP335 or a truncated version that lacks the TMD (LysP335ΔTMD) in the expression vector pETG-20A. The whole gene or part of was amplified using the specific primer pairs listed in S1 Table and subcloned in pETG-20A using Gateway assembly. Subsequent plasmids named pLysP335^20a^ and pLysP335ΔTMD^20a^ were transformed in One Shot BL21(DE3) chemically competent *E*. *coli* cells and propagated on LB plates or broth supplemented with ampicillin. All constructs were validated by DNA sequencing using primers listed in S1 Table.

For protein induction, *E*. *coli* cells were grown in TB medium at 37°C until an optical density of 0.6 to 0.8 (OD_600nm_) was reached. After induction with 1 mM isopropyl-β-D-1-thiogalactopyranoside (IPTG), cells were further cultured for another 16 h at 18°C. For LysP008, Lysc2, and Lys1358 purification, induced cells were resuspended in lysis buffer (50 mM Tris–HCL (pH 8.0), 300 mM NaCl, 5% glycerol, 5 mM imidazole, 1 mM PMSF) and were sonicated and centrifuged. Supernatants were then subjected to nickel affinity chromatography and the target proteins were eluted with buffer containing 200 mM imidazole. Eluted proteins were then loaded on a Superdex 200 Increase (10/300GL) column for further purification and sample buffer exchange (20 mM Tris–HCL (pH 8.0), 150 mM NaCl, 1% glycerol, 1 mM DTT).

LysP335 and LysP335ΔTMD were purified from inclusion bodies (IBs). Cells were first sonicated in a lysis buffer containing 50 mM Tris–HCL, 300 mM NaCl, 2 mM EDTA, 5% saccharose, 30 mg/L DNase, and 30 mg/L fresh RNase (pH 8.0). IBs were isolated using a 20-min centrifugation step at 4°C (8,000 rpm) and then washed twice with a buffer containing 50 mM Tris–HCL and 2 mM EDTA (pH 8.0) for LysP335. For P335ΔTDM, we used the same buffer, plus 0.1% Triton X-100. A second washing step was performed with a buffer containing 50 mM Tris–HCL and 1 M urea (pH 8.0). Then, IBs were solubilized with 10 ml of denaturing buffer (50 mM Tris–HCL (pH 8.0), 5 mM EDTA, 0.15 M NaCl, and 6 M guanidinium hydrochloride). For refolding, we diluted the solution containing denatured proteins with refolding buffer (50 mM Tris–HCL (pH 8.0), 0.5 M L-arginine, 0.1 M NaCl, and 0.01% Brij-35) 20 times at a flow rate of 0.5 ml/min at 4°C. Refolded proteins were concentrated to 50 mL and dialyzed overnight at 4°C with a buffer containing 50 mM Tris–HCL, 300 mM NaCl, 5% glycerol, and 5 mM imidazole (pH 8.0). One-step purification by nickel affinity chromatography was carried out as described for the other endolysins.

### Evaluation of endolysin activity on *L*. *lactis* cells and peptidoglycan

The lytic activity of the purified endolysins was measured by following the decrease in turbidity of a solution containing *L*. *lactis* IL1403 cells in exponential growth phase (0.4 OD_600nm_). A volume of 150 μl of bacterial cells resuspended in lysis buffer (40 mM phosphate buffer, 200 mM NaCl (pH 8.0)) was mixed in a 96-well microplate with 150 μl of purified endolysin at the desired concentration. The decrease in turbidity was recorded using a microplate reader. For glucosamidase activity assessment, peptidoglycan was extracted from *L*. *lactis* IL1403 as described previously [45] and resuspended in water to reach a final concentration of 10 mg/mL. Enzymatic digestion was achieved by mixing 100 μl of the extracted peptidoglycan with 900 μl of the previously purified endolysin at a final concentration of 3.5 μM. After an overnight incubation at 37°C with agitation, the solution was heated for 3 min at 100°C to inactivate the endolysin. Next, the solution was centrifuged at 13,000 rpm for 10 min. The supernatant containing the digested peptidoglycan was analyzed using a modified Park–Johnson assay [46]. Undigested peptidoglycan was used as a blank and glucose was used to calibrate the curve.

### Phage P335 and P008 genome editing

Spacers targeting the genes that code for phage P335 and P008 endolysins were cloned into the crRNA of plasmid pL2Cas9 using oligonucleotide pairs pL2Cas9_LysP335_5′/pL2Cas9_LysP335_3′ and pL2Cas9_LysP008_2_5′/pL2Cas9_LysP008_2_3′, as described previously [47]. Plasmids pL2Cas9-LysP335 and pL2Cas9-LysP008 were then transformed in *L*. *lactis* IL1403 and the correct spacers were confirmed using the sequences of the PCR products obtained with primers crRNA_S.pyo_R and Cas9_S.pyo_F6.

The different endolysins genes were directly swapped by keeping the original start and stop codon of the phage P335 and P008 endolysins. To this end, repair templates composed of the new endolysin flanked by 2 ca. 250-bp arms that share homology with DNA sequences upstream and downstream of *LysP335* or *LysP008* were designed. These gene fragments were synthetized with complementary ends (Integrated DNA Technologies (IDT)) for Gibson assembly at the XbaI restriction site of plasmid pNZ123. For non-lactococcal phage endolysin genes, additional codon optimization for *L*. *lactis* IL1403 was introduced. The sequences of the inserts in pNZ123 were confirmed through PCR analysis of the colony with primers pNZins_F and pNZins_R and subsequent sequencing. Similarly, the repair templates used to delete the 2 LysM CBDs of LysP335 were composed of a truncated version of the *LysP335* flanked by 2 ca. 250-bp arms that share homology with DNA sequences upstream and downstream of *LysP335*. This fragment was further cloned in pNZ123, as described previously.

Recombinant phages were isolated from plaques after infection of *L*. *lactis* IL1403 strains that were transformed with the pL2Cas9 and repair template plasmids. Isolated phage plaques were analyzed by PCR with primer pairs P335_CONTROL_F / P335_CONTROL_R for phage P335 or P008_CONTROL_F / P008_CONTROL_R for phage P008. Modified phages with the correct mutation were further sequenced.

### One-step growth curves, replication dynamics, and plaque size analysis

Phage growth curves were determined at 30°C with a starting multiplicity of infection (MOI) of 0.05, as described previously [48]. To determine replication dynamics of the phage P335 and endolysins mutants, *L*. *lactis* IL6228 cells in the exponential growth phase (OD_600nm_ 0.2) were infected at an initial MOI of 0.000001 for 18 h at 30°C. The phage–bacteria population was then diluted to 1/100-fold and transferred to a new tube until clear lysis of the culture was observed. The surface of the lytic plaques produced by each of the phage mutants was inferred from image analysis of plates using a fixed focal camera. A millimetric ruler was superimposed on the plates to assess the number of pixels per mm. Using imageJ, lytic plaques were manually overlaid, and the threshold option was used to extract the surface for further particle size analysis (see S13 Fig for an example with phage P335).

### Phylogenetic analysis of lactococcal virulent and temperate phages as well as and prophages

Prophage sequences were retrieved from 26 fully annotated *L*. *lactis* genomes using PHASTER [49]. Mash (v2.2) was used for simple distance estimation [50] and the output was used to produce a phylip-formatted square matrix. Rapidnj (v2.3.2) was used for neighbor joining [51]. The phylogenetic trees were visualized and rerooted using the iTOL v5 web interface [40].

### Analysis of homology between genomes and endolysins from phages infecting *L*. *lactis* and other bacterial species

The NCBI Virus database was downloaded (October 27, 2020) in nucleotide and protein formats for all phages (*n =* 19,201), and custom blast databases were generated. The RefSeq prokaryotes reference genomes (ref_prok_rep_genomes) and RefSeq protein (refseq_protein) databases were also downloaded from NCBI to perform local homology searches. To search for nucleotide matches, blastn (v2.9.0) was used with the default parameters, and we queried for lactococcal phage genomes [52]. To search for protein matches, blastp (v2.9.0) was used with lactococcal phage endolysins as the query term. To limit the homology search to bacterial proteins, the get_species_taxids.sh BLAST Command Line Applications script was used to obtain a list of taxonomic IDs indicating bacterial origin. Then, the -taxidlist parameter was added to the blastp command to specify bacterial taxonomic IDs. Nucleotide hits were filtered, retaining hits with an alignment length ≥1,000 bp. Protein hits were also filtered, retaining hits with a query coverage ≥90% and a percent identity ≥50%.

### Experimental evolution of the different phage mutants generated during the study

Endolysin mutants from phages P335 and P008 were successively amplified for a total of 20 transfers. *L*. *lactis* IL6228 cells in the exponential growth phase (OD_600nm_ 0.2) were infected at an initial MOI of 10. After an incubation period of 12 h at 30°C, phage lysates were filtered and diluted before another amplification round was started. Dilutions of 1/1,000 were used for phages P335, P335>LysP008, P335>Lysc2, P335>Lys1358, P008>LysEFAS, P008>LysFL3B, and P008>LysLfeSau and dilutions of 1/10,000 for phages P008, P008>LysP335, P008>Lysc2, and P008>Lys1358.

### Phage DNA sequencing and analysis

Phage genomic DNA was extracted as described elsewhere [53], and libraries were prepared using the Nextera XT DNA library preparation kit (Illumina) according to the manufacturer’s instructions. Sequencing was performed on a MiSeq system using a MiSeq reagent kit v2 (Illumina). The reads were cleaned using Trimmomatic v0.36 [54] and assembled to obtain circular complete sequences using Ray v3.0.1 [55] and SPAdes v3.13 [56].

## Supporting information

S1 Fig Multiple sequence alignment of CHAP CDs from lactococcal phage endolysins belonging to cluster B. Similar residues are colored according to their level of conservation based on BLOSUM62 scores (100% similar: black; 80% to 100%: gray; 60% to 80% light gray; less than 60%: white). The identity over all pairs in the column is indicated on the top (green: 100% identity, green-brown: between 30%, and 100% identity, red: below 30% identity). The conserved cysteine and histidine residues that are part of the CHAP active site are highlighted in red. The alignment and figure were generated using Geneious v11.1.5 [57]. CD, catalytic domain.
(TIF)

S2 Fig **Identification of TMDs (A) and SP (B) in the endolysin of the virulent lactococcal phage P335**. Predictions for the presence of a TMD were performed using the TMHMM tool [43]. SP and CS were identified using the SignalP 5.0 server [44]. CS, cleavage site; SP, signal peptide; TMD, transmembrane domain.
(TIF)

S3 Fig Phylogenetic relationships of the holins observed in lactococcal phages. We investigated the diversity of holins found in 253 complete lactococcal phage genomes available in GenBank. ClustalW (v2.1) was used to perform multiple alignments and generate a phylogenetic tree (S7 Data). The holin class was identified according to the number of TMDs using the TMHMM tool (see also S4 Fig and S7 Data) [43]. The name of the phage is indicated, followed by the accession number of its respective endolysin. CBD, cell wall–binding domain; CD, catalytic domain; TMD, transmembrane domain.
(TIF)

S4 Fig Examples of TMD identification for each holin group observed in S3 Fig. Predictions of TMDs were performed using the TMHMM tool (S7 Data) [43]. TMD, transmembrane domain.
(TIF)

S5 Fig SDS-PAGE gel showing the purified endolysins used in the study. Proteins were loaded on NuPAGE 4%–12% BisTris gels and stained with Coomassie Blue. The expected molecular mass of each purified protein is indicated on the top of the figure. Molecular weight markers are on the left.
(TIF)

S6 Fig Identification of alternative stop codons and internal ribosomal binding sites in LysP008, Lysc2, and related endolysins. **(**A) Schematic representation of the LysP008 endolysins domains and its alternative start codon (M 172) observed at the beginning of EME_EF1-like CBD. The 25-bp present before the alternative start codon (in blue) compared to a consensus sequence generated with Weblogo [58] and 14 related endolysins (Phages Q54 (YP_762603.1), p2 (ADC80094.1), sk1 (NP_044966.1), 712 (YP_764281.1), 56301 (ASZ71451.1), ASCC287 (AFE86988.1), 936 (AGI10761.1), LP0509 (ATE84099.1), 66901 (ASZ71714.1), 17W12M (AOQ30170.1), 38503 (ASZ71276.1), ASCC473 (AFE86646.1), Phi19 (ALM63160.1)) (S7 Data). (B) Schematic representation of the Lysc2 endolysins domains and its putative alternative start codon (M 156) observed at the beginning of EME_EF1-like CBD. The 25-bp present before the alternative start codon (in blue) are compared to a consensus sequence based on 9 related endolysins (Phages 62402 (ASZ70696.1), 37203 (ASZ70771.1), bIL67 (NP_042321.2), 62606 (ASZ70659.1), 62403 (ASZ70733.1), 05802 (ASZ70922.1), 50504 (ASZ70846.1), 50102 (ASZ70884.1), 20R03M (QBQ82006.1)) (S7 Data). The putative internal ribosomal binding site sequence is underlined in red. CBD, cell wall–binding domain.
(TIF)

S7 Fig Expression of lysc2 and LysP008 without codon optimization. **(**A) Alignment of the 25-bp present before the internal start codon of the *LysP008* and *Lysc2* genes with or without codon optimization for *E*. *coli* BL21. (B) Purification of the LysP008 and Lysc2 without codon optimization and size exclusion chromatography (2 liters cell culture).
(TIF)

S8 Fig Identification of bacterial species infected by phages with endolysins or genomes that share homology with lactococcal phages. **(**A) Analysis of non-lactococcal phages that have endolysins with at least 90% coverage and 50% identity to endolysins of lactococcal phages (S7 Data). B) Analysis of non-lactococcal phage genomes with at least 1,000-bp homology with lactococcal phages. *L*. *lactis* phages are grouped according to their type of endolysin (S7 Data). CD, catalytic domain.
(TIF)

S9 Fig Phylogenetic relationship of endolysins of virulent or temperate phages infecting *Enterococcus faecalis*. We investigated the diversity of endolysins found in 70 complete genomes of phages that infect *E*. *faecalis*. ClustalW (v2.1) was used to perform multiple alignments and generate a phylogenetic tree (S7 Data). Conserved CD and CBD were determined according to HHPred and BLASTP. The type of domain and accession number is indicated for each type of endolysin. CBD, cell wall–binding domain; CD, catalytic domain.
(TIF)

S10 Fig Phylogenetic relationship of endolysins of virulent or temperate phages infecting *Limosilactobacillus fermentum* (formely *Lactobacillus fermentum*). We investigated the diversity of endolysins found in 3 complete genomes from phages infecting *L*. *fermentum*. ClustalW (v2.1) was used to perform multiple alignments and generate a phylogenetic tree (S7 Data). Conserved CD and CBD were determined according to HHPred and BLASTP. The type of domain and accession number is indicated for each type of endolysin. CBD, cell wall–binding domain; CD, catalytic domain.
(TIF)

S11 Fig Phylogenetic relationship of endolysins of virulent or temperate phages infecting *Streptococcus thermophilus*. We investigated the diversity of endolysins found in 56 complete genomes from phages infecting *S*. *themophilus*. ClustalW (v2.1) was used to perform multiple alignments and generate a phylogenetic tree (S7 Data). Conserved CD and CBD were determined according to HHPred and BLASTP. The type of domain and accession number is indicated for each type of endolysin. CBD, cell wall–binding domain; CD, catalytic domain.
(TIF)

S12 Fig Genomic alignment of the lactococcal phages bIL285, P335, and mutants after 10 (T10) and 20 (T20) transfers. Recombination between the prophage bIL285 and the virulent phage P335 was observed after either 10 or 20 transfers. A cutoff of 95% identity was used for the genome’s alignment. Homology regions were recombination between the phage P335 and prophage BIL285 take place are highlighted in red.
(TIF)

S13 Fig Example of lytic plaques surface quantification of the lactococcal phage P335. **(**A) Image of the phage P335 plaques at a resolution of 31 pixels per mm. (B) Using ImageJ [59], lytic plaques were manually overlaid, and (C) the threshold option was used to subtract the image background. (D). The analyse particles command was finally used to measure the plaques surface in the thresholded image.
(TIF)

S1 Table Mutations in the phage genomes during the experimental evolution assays. (DOCX)

S2 Table Bacterial strains, plasmids, and nucleotides. (DOCX)

S1 Data Individual numerical values that underlie the data summarized in Figs 1, 2B–2D, 3C, 4A–4C, 5A and 5D, 6A and 6C, and 7B–7E. (XLSX)

S2 Data Individual numerical values that underlie the data summarized in Fig 8A. (XLSX)

S3 Data Individual numerical values that underlie the data summarized in Fig 8B. (XLSX)

S4 Data Individual numerical values that underlie the data summarized in Fig 8C. (XLSX)

S5 Data Individual numerical values that underlie the data summarized in Fig 8D. (XLSX)

S6 Data Individual numerical values that underlie the data summarized in Fig 8E. (XLSX)

S7 Data Individual numerical values that underlie the data summarized in S3, S4, S6A and S6B, S8A and S8B, S9, S10, and S11 Figs. (XLSX)

## Abbreviations

CBD: cell wall–binding domain
CD: catalytic domain
CS: cleavage site
IB: inclusion body
iTOL: Interactive Tree Of Life
MOI: multiplicity of infection
PCR: polymerase chain reaction
TMD: transmembrane domain
