# Supplementary figures and images for: Phage endolysins are adapted to specific hosts and are evolutionarily dynamic
Source: PLoS Biol. 2022 Aug 1;20(8):e3001740. doi: 10.1371/journal.pbio.3001740 (PMC9371310; doi:10.1371/journal.pbio.3001740)

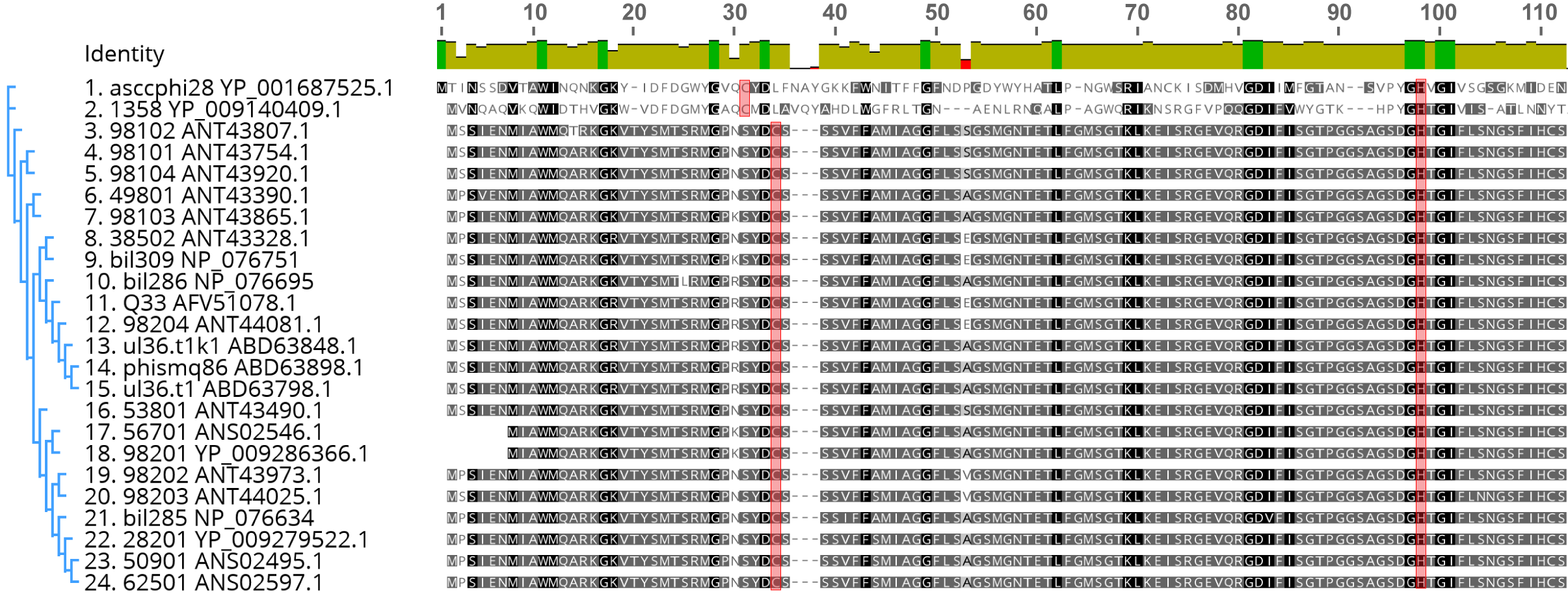

Supplement: S1 Fig — Similar residues are colored according to their level of conservation based on BLOSUM62 scores (100% similar: black; 80% to 100%: gray; 60% to 80% light gray; less than 60%: white). The identity over all pairs in the column is indicated on the top (green: 100% identity, green-brown: between 30%, and 100% identity, red: below 30% identity). The conserved cysteine and histidine residues that are part of the CHAP active site are highlighted in red. The alignment and figure were generated using Geneious v11.1.5 [57]. CD, catalytic domain. (TIF) [file pbio.3001740.s001.tif]

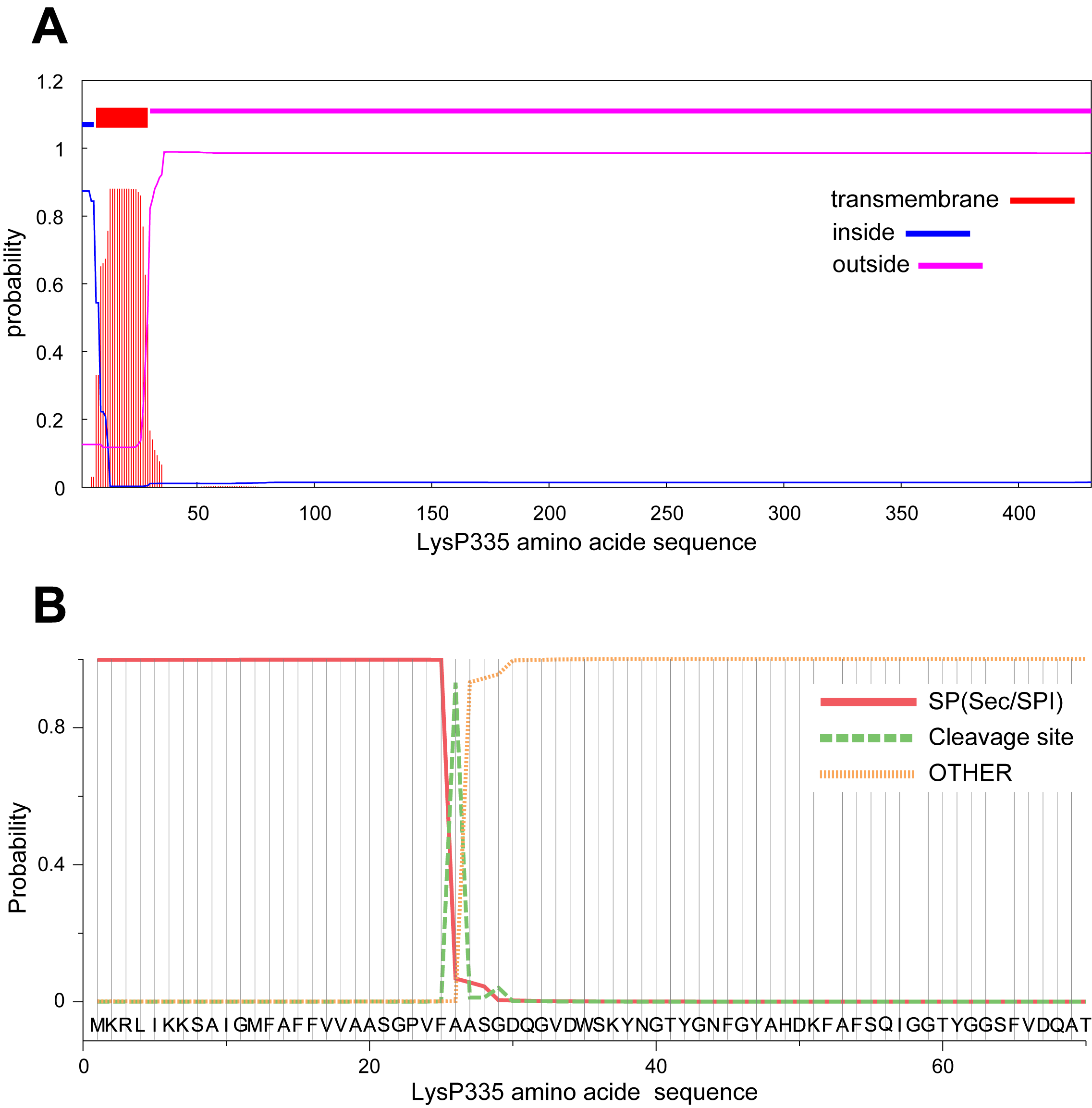

Supplement: S2 Fig — Identification of TMDs (A) and SP (B) in the endolysin of the virulent lactococcal phage P335. Predictions for the presence of a TMD were performed using the TMHMM tool [43]. SP and CS were identified using the SignalP 5.0 server [44]. CS, cleavage site; SP, signal peptide; TMD, transmembrane domain. (TIF) [file pbio.3001740.s002.tif]

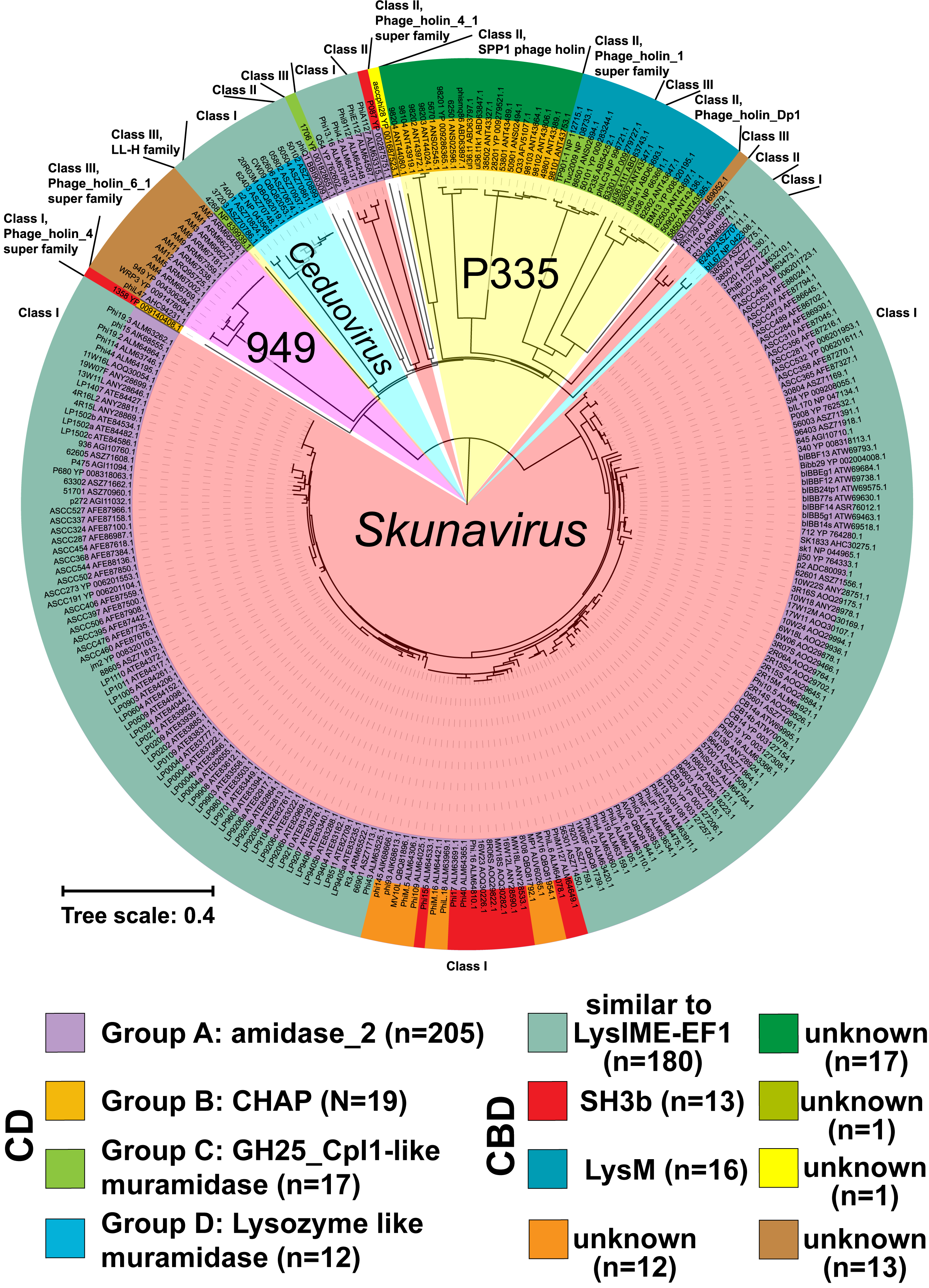

Supplement: S3 Fig — We investigated the diversity of holins found in 253 complete lactococcal phage genomes available in GenBank. ClustalW (v2.1) was used to perform multiple alignments and generate a phylogenetic tree (S7 Data). The holin class was identified according to the number of TMDs using the TMHMM tool (see also S4 Fig and S7 Data) [43]. The name of the phage is indicated, followed by the accession number of its respective endolysin. CBD, cell wall–binding domain; CD, catalytic domain; TMD, transmembrane domain. (TIF) [file pbio.3001740.s003.tif]

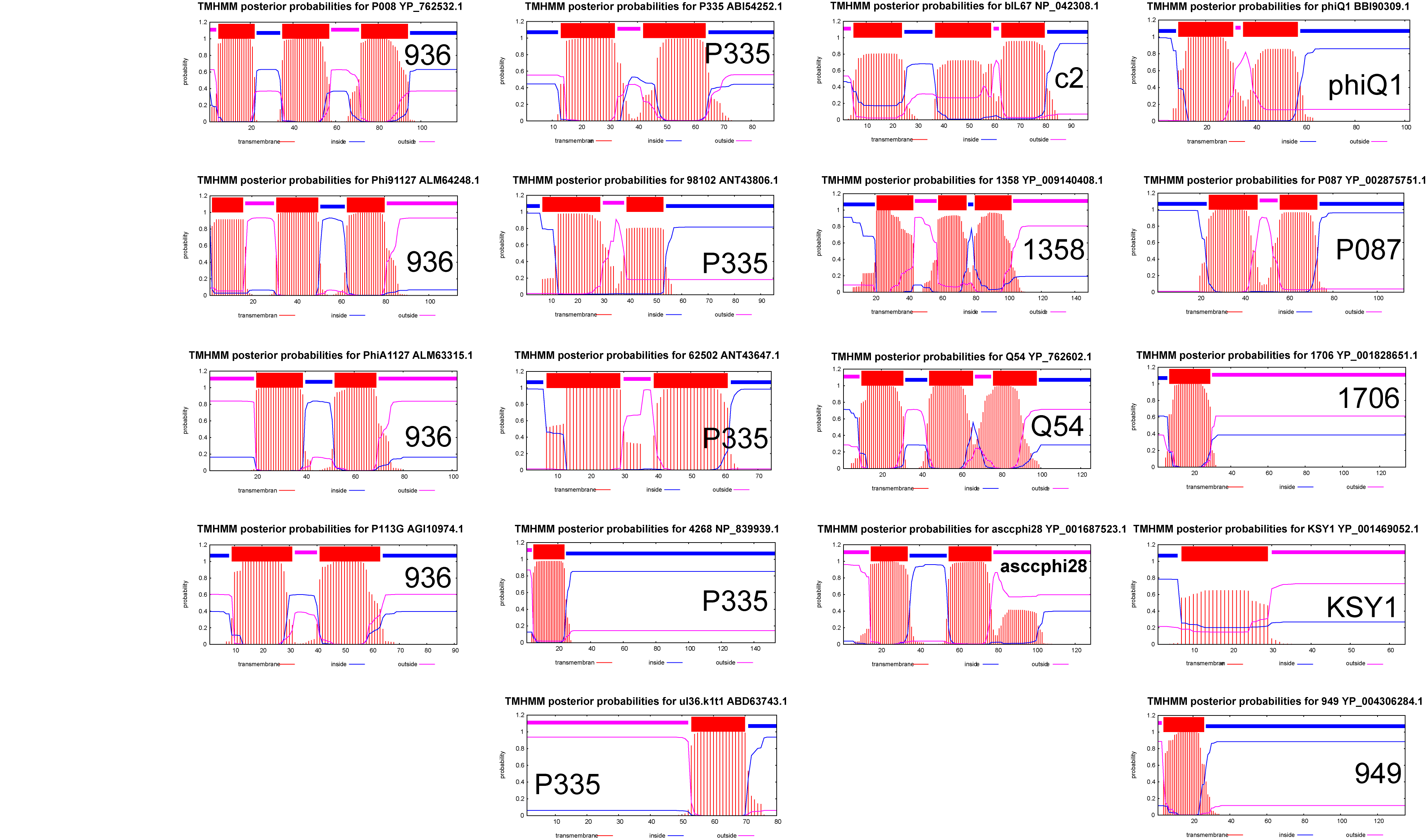

Supplement: S4 Fig — Predictions of TMDs were performed using the TMHMM tool (S7 Data) [43]. TMD, transmembrane domain. (TIF) [file pbio.3001740.s004.tif]

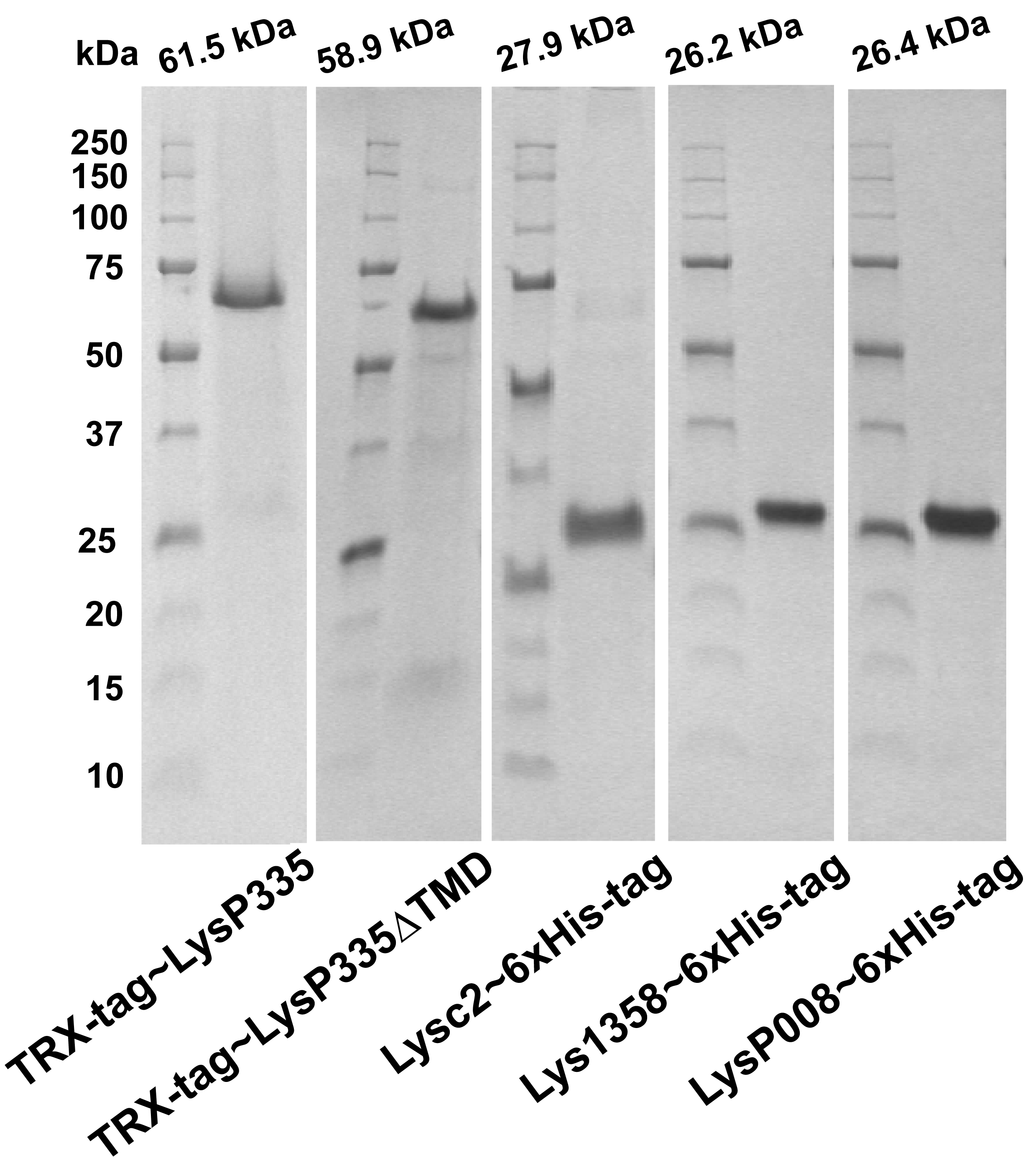

Supplement: S5 Fig — Proteins were loaded on NuPAGE 4%–12% BisTris gels and stained with Coomassie Blue. The expected molecular mass of each purified protein is indicated on the top of the figure. Molecular weight markers are on the left. (TIF) [file pbio.3001740.s005.tif]

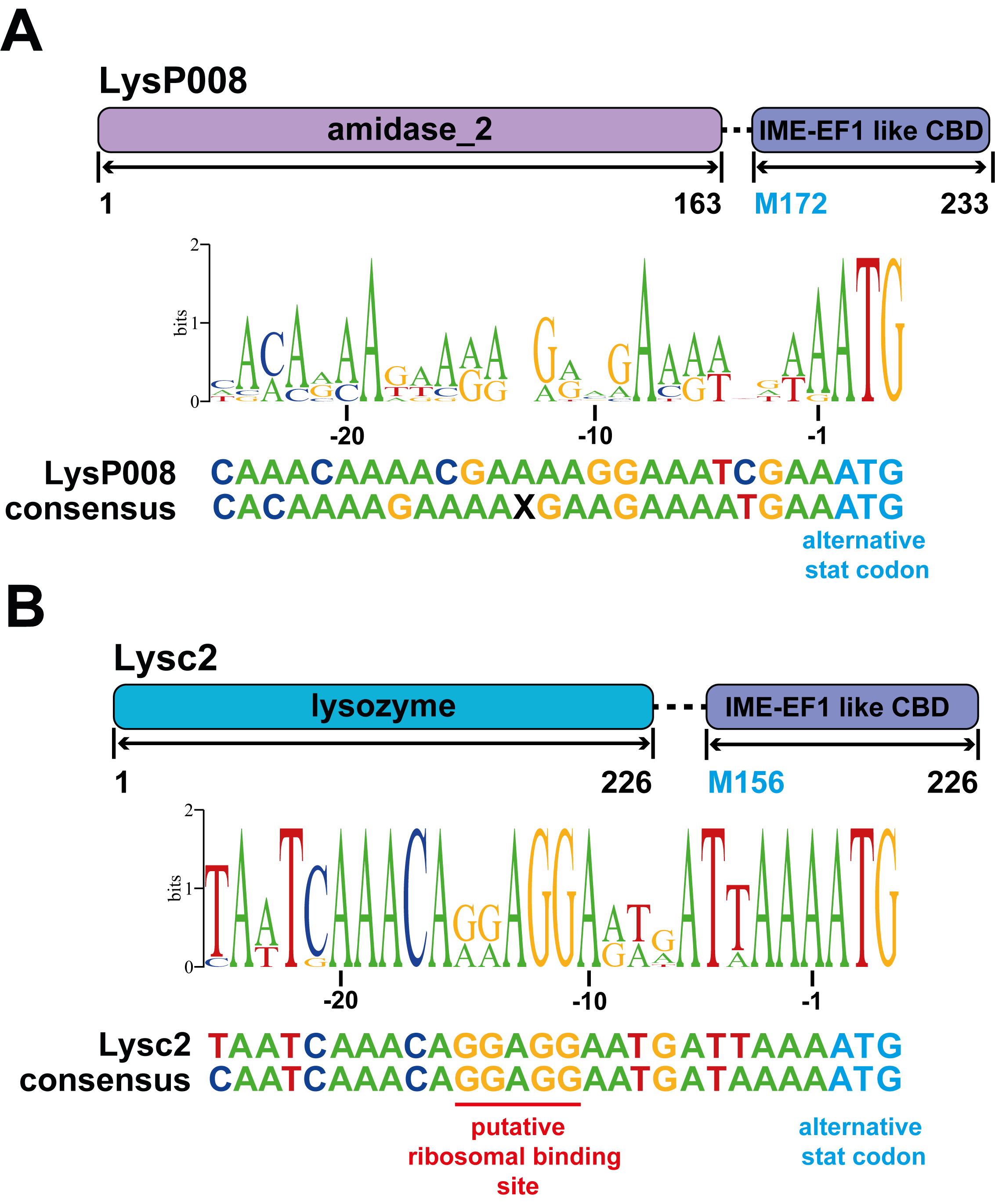

Supplement: S6 Fig — (A) Schematic representation of the LysP008 endolysins domains and its alternative start codon (M 172) observed at the beginning of EME_EF1-like CBD. The 25-bp present before the alternative start codon (in blue) compared to a consensus sequence generated with Weblogo [58] and 14 related endolysins (Phages Q54 (YP_762603.1), p2 (ADC80094.1), sk1 (NP_044966.1), 712 (YP_764281.1), 56301 (ASZ71451.1), ASCC287 (AFE86988.1), 936 (AGI10761.1), LP0509 (ATE84099.1), 66901 (ASZ71714.1), 17W12M (AOQ30170.1), 38503 (ASZ71276.1), ASCC473 (AFE86646.1), Phi19 (ALM63160.1)) (S7 Data). (B) Schematic representation of the Lysc2 endolysins domains and its putative alternative start codon (M 156) observed at the beginning of EME_EF1-like CBD. The 25-bp present before the alternative start codon (in blue) are compared to a consensus sequence based on 9 related endolysins (Phages 62402 (ASZ70696.1), 37203 (ASZ70771.1), bIL67 (NP_042321.2), 62606 (ASZ70659.1), 62403 (ASZ70733.1), 05802 (ASZ70922.1), 50504 (ASZ70846.1), 50102 (ASZ70884.1), 20R03M (QBQ82006.1)) (S7 Data). The putative internal ribosomal binding site sequence is underlined in red. CBD, cell wall–binding domain. (TIF) [file pbio.3001740.s006.tif]

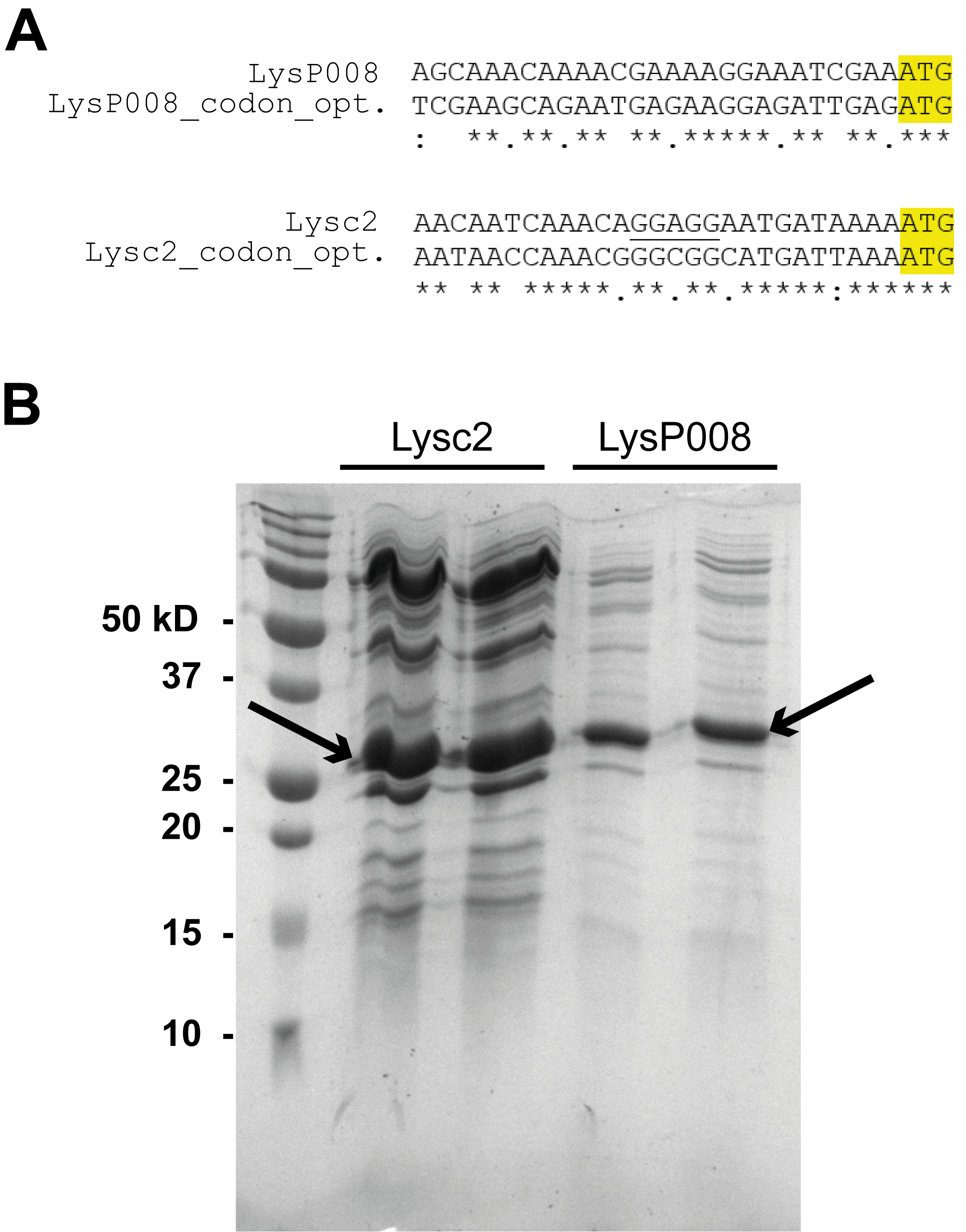

Supplement: S7 Fig — (A) Alignment of the 25-bp present before the internal start codon of the LysP008 and Lysc2 genes with or without codon optimization for E. coli BL21. (B) Purification of the LysP008 and Lysc2 without codon optimization and size exclusion chromatography (2 liters cell culture). (TIF) [file pbio.3001740.s007.tif]

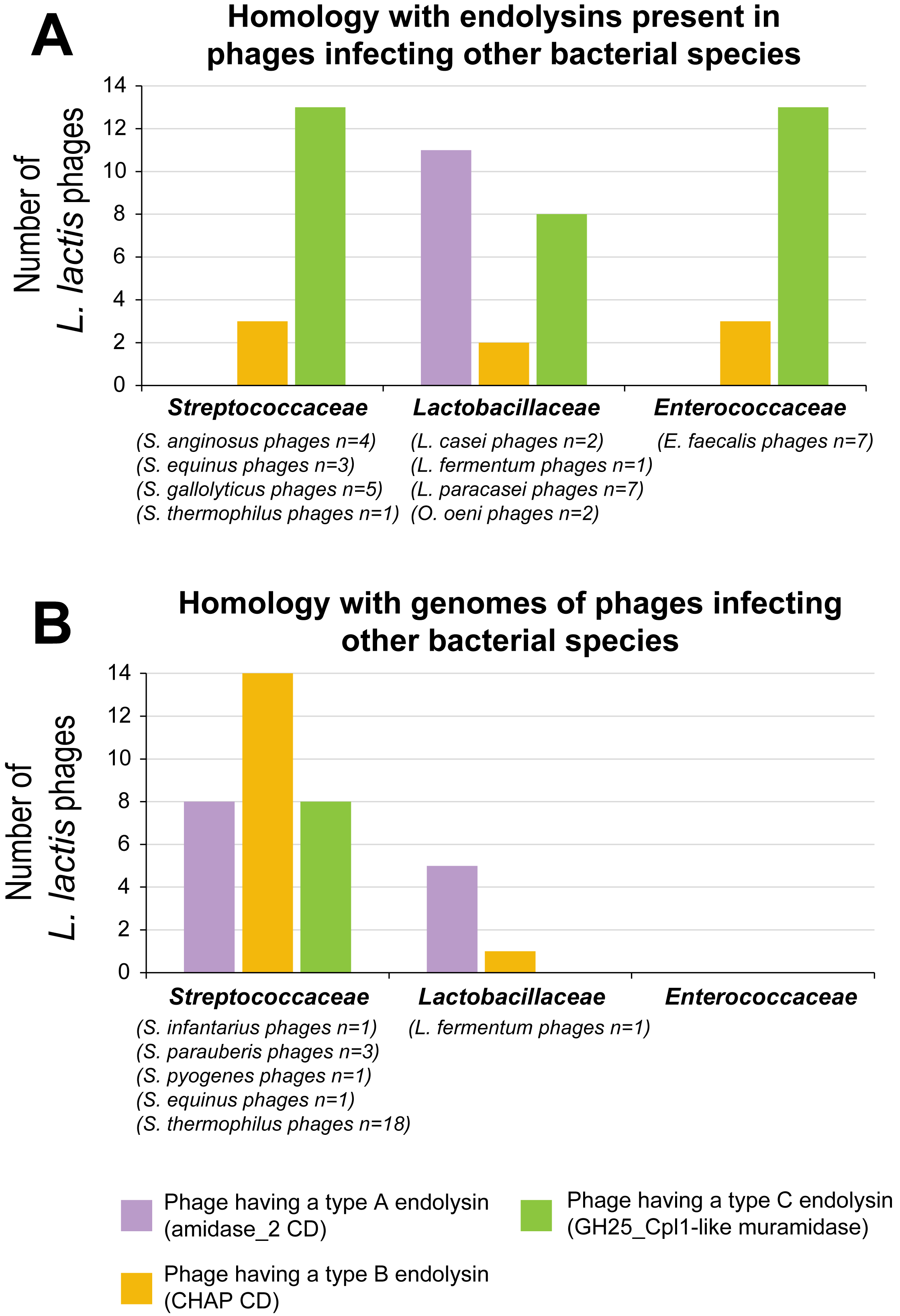

Supplement: S8 Fig — (A) Analysis of non-lactococcal phages that have endolysins with at least 90% coverage and 50% identity to endolysins of lactococcal phages (S7 Data). B) Analysis of non-lactococcal phage genomes with at least 1,000-bp homology with lactococcal phages. L. lactis phages are grouped according to their type of endolysin (S7 Data). CD, catalytic domain. (TIF) [file pbio.3001740.s008.tif]

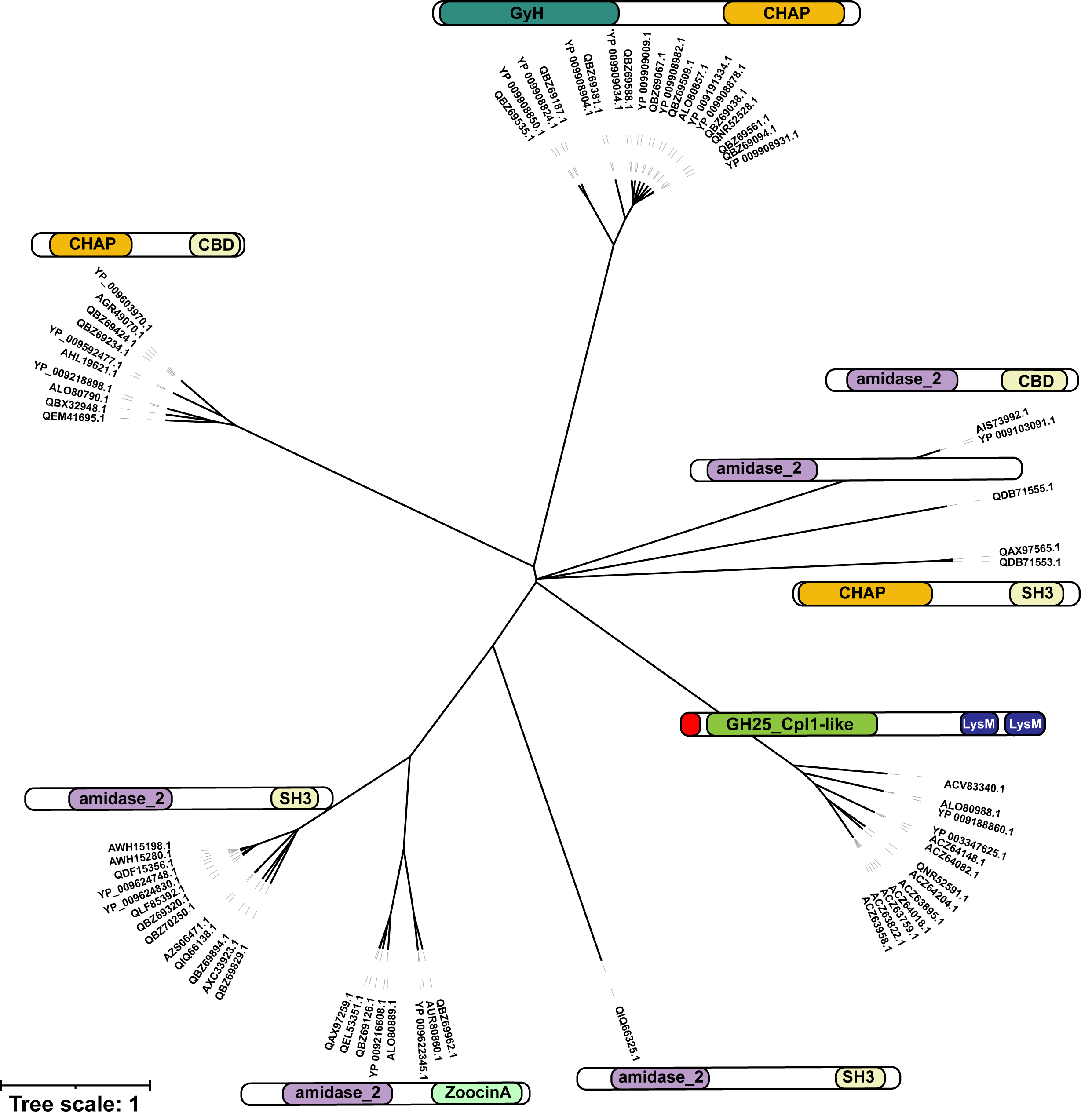

Supplement: S9 Fig — We investigated the diversity of endolysins found in 70 complete genomes of phages that infect E. faecalis. ClustalW (v2.1) was used to perform multiple alignments and generate a phylogenetic tree (S7 Data). Conserved CD and CBD were determined according to HHPred and BLASTP. The type of domain and accession number is indicated for each type of endolysin. CBD, cell wall–binding domain; CD, catalytic domain. (TIF) [file pbio.3001740.s009.tif]

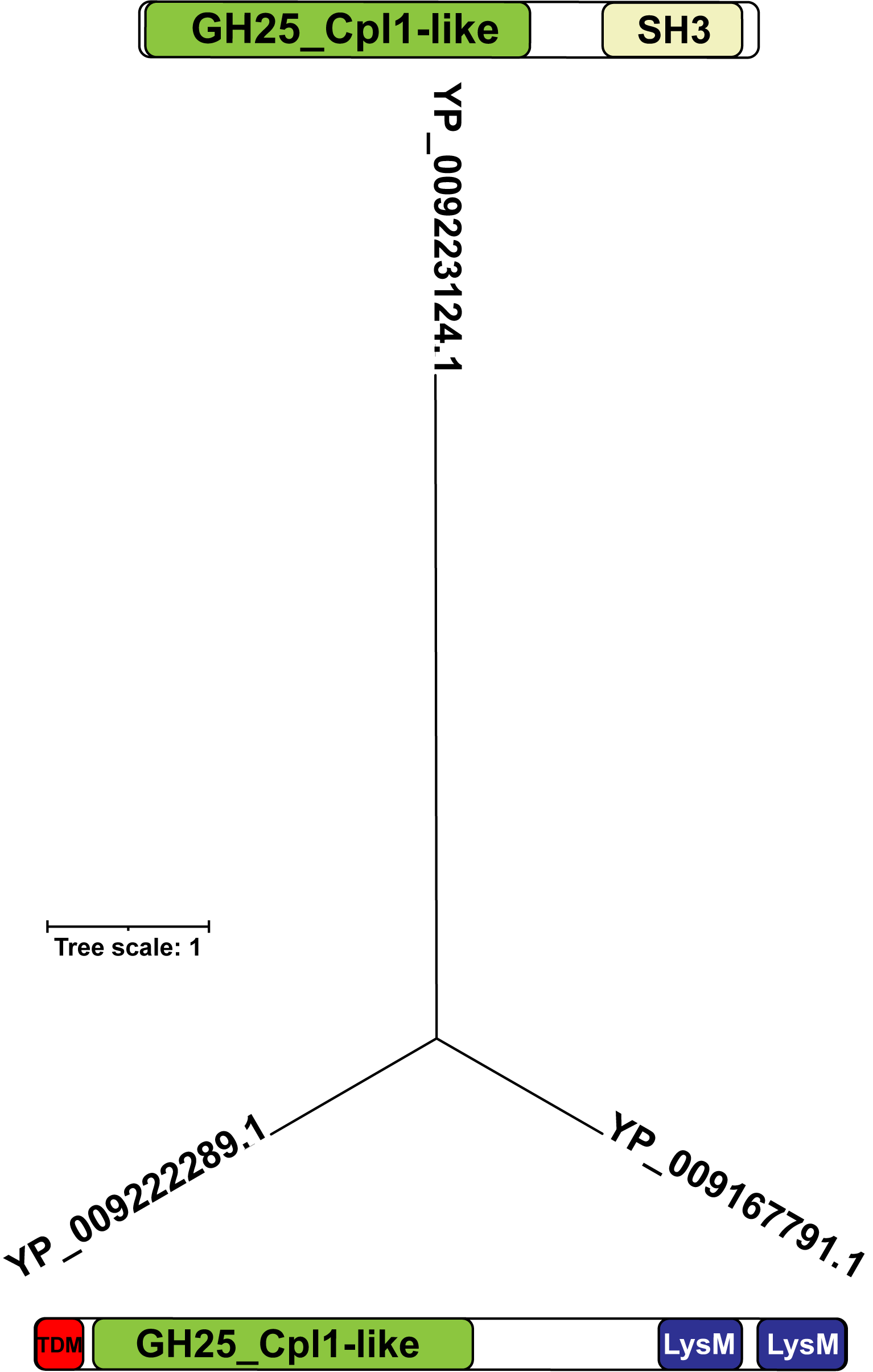

Supplement: S10 Fig — We investigated the diversity of endolysins found in 3 complete genomes from phages infecting L. fermentum. ClustalW (v2.1) was used to perform multiple alignments and generate a phylogenetic tree (S7 Data). Conserved CD and CBD were determined according to HHPred and BLASTP. The type of domain and accession number is indicated for each type of endolysin. CBD, cell wall–binding domain; CD, catalytic domain. (TIF) [file pbio.3001740.s010.tif]

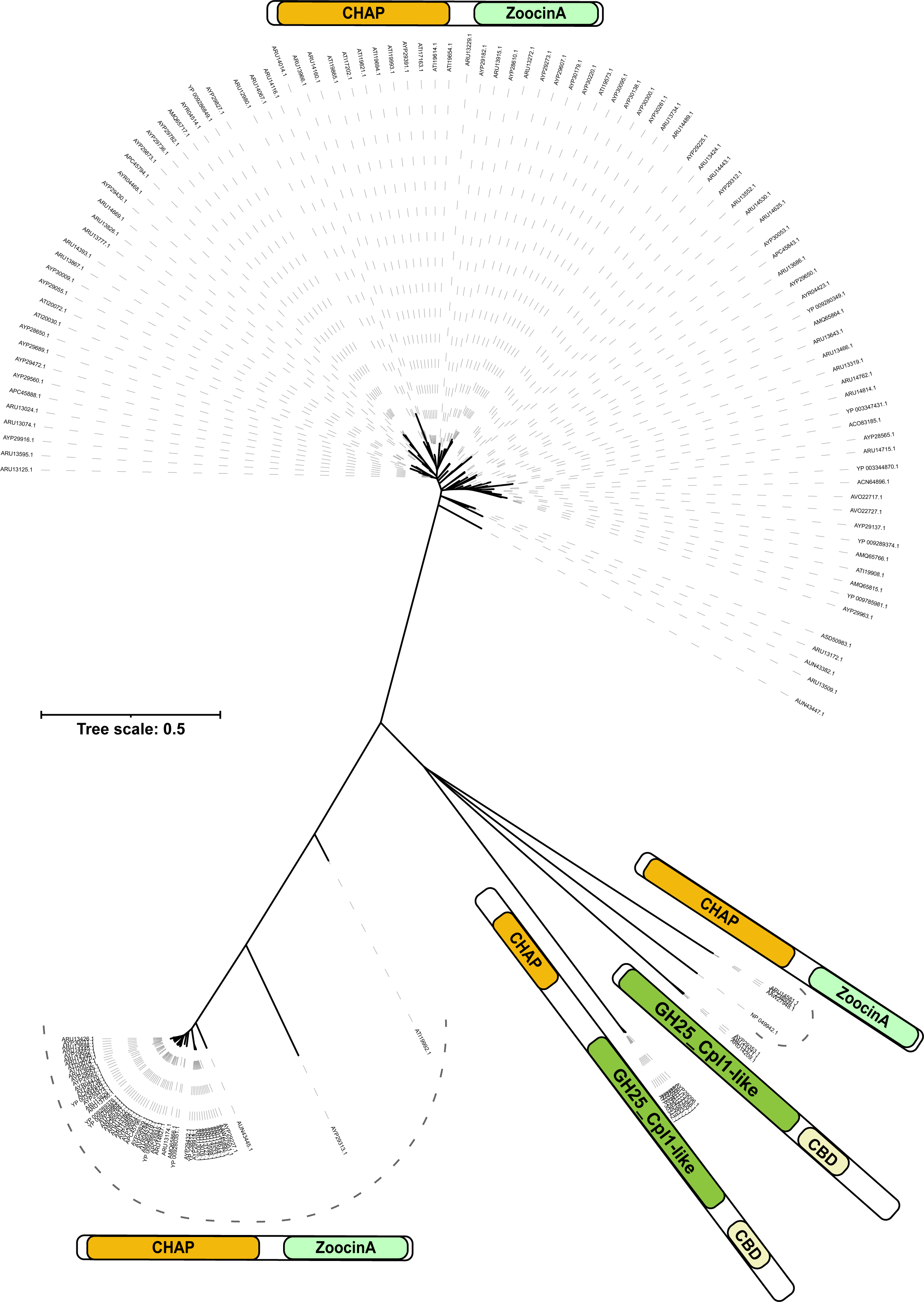

Supplement: S11 Fig — We investigated the diversity of endolysins found in 56 complete genomes from phages infecting S. themophilus. ClustalW (v2.1) was used to perform multiple alignments and generate a phylogenetic tree (S7 Data). Conserved CD and CBD were determined according to HHPred and BLASTP. The type of domain and accession number is indicated for each type of endolysin. CBD, cell wall–binding domain; CD, catalytic domain. (TIF) [file pbio.3001740.s011.tif]

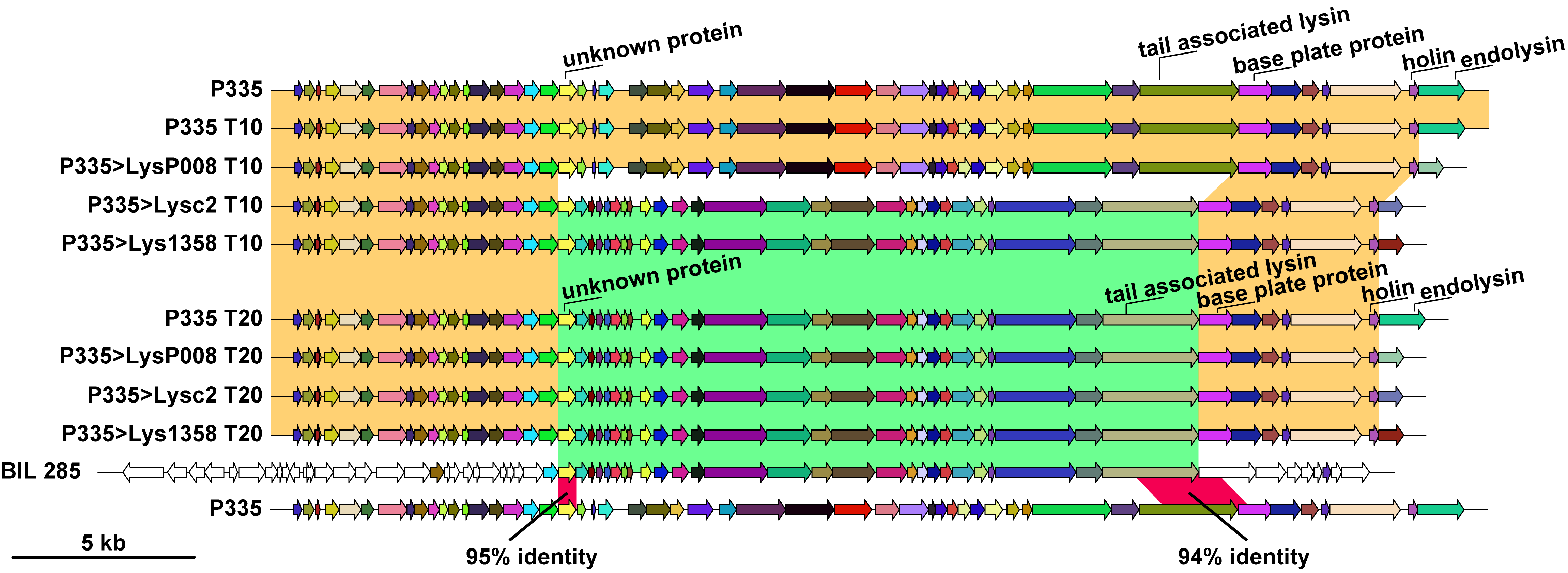

Supplement: S12 Fig — Recombination between the prophage bIL285 and the virulent phage P335 was observed after either 10 or 20 transfers. A cutoff of 95% identity was used for the genome’s alignment. Homology regions were recombination between the phage P335 and prophage BIL285 take place are highlighted in red. (TIF) [file pbio.3001740.s012.tif]

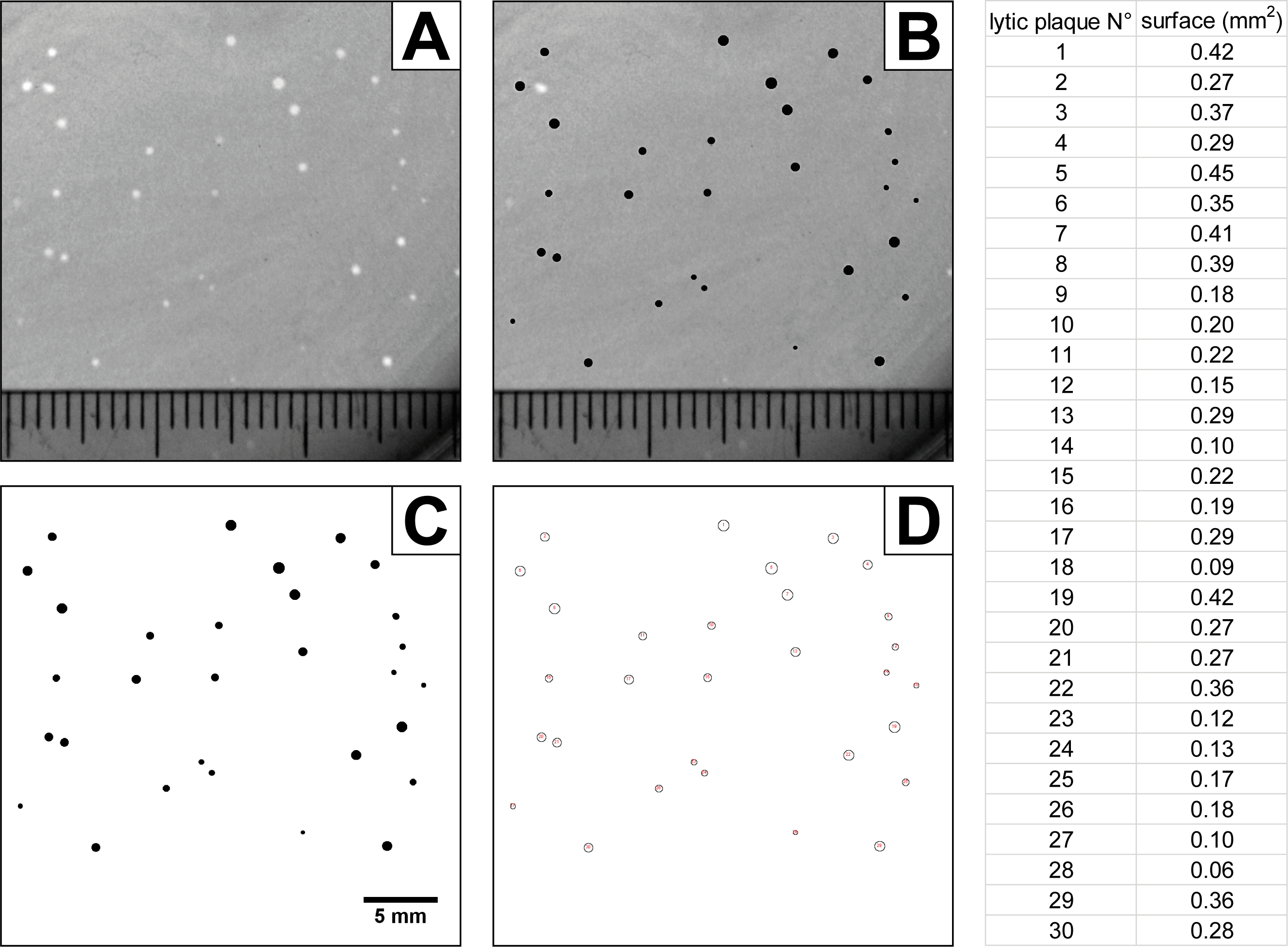

Supplement: S13 Fig — (A) Image of the phage P335 plaques at a resolution of 31 pixels per mm. (B) Using ImageJ [59], lytic plaques were manually overlaid, and (C) the threshold option was used to subtract the image background. (D). The analyse particles command was finally used to measure the plaques surface in the thresholded image. (TIF) [file pbio.3001740.s013.tif]
