# Supplementary material for: Phage endolysins are adapted to specific hosts and are evolutionarily dynamic
Source: PLoS Biol. 2022 Aug 1;20(8):e3001740. doi: 10.1371/journal.pbio.3001740 (PMC9371310; doi:10.1371/journal.pbio.3001740)
Supplement: S1 Table — (DOCX) [file pbio.3001740.s014.docx]

|  | P1 |  | P10 |  | P20 |  |  |  |
| --- | --- | --- | --- | --- | --- | --- | --- | --- |
|  | nt | AA | nt | AA | nt | AA | |  |
| **P008 WT** | 9072, A ->T | R->S | 9072, A ->T | R->S | 9072, A ->T | R->S | mutation 1 | TMP |
|  | 9943, A->G | G->S | 9943, A->G | G->S | 9943, A->G | G->S | mutation 2 | TMP |
|  |  |  | 9080, C->A | T->K | 9080, C->A | T->K | mutation 3 | TMP |
|  |  |  | 14900, T->C | V->A | 14900, T->C | V->A | mutation 4 | BP |
|  |  |  |  |  | 14672, G->T | R->I | mutation 5 | BP |
|  |  |  |  |  |  |  |  |  |
| **P008>LysP335** | 9072, A ->T | R->S | 9072, A ->T | R->S | 9072, A ->T | R->S | mutation 1 | TMP |
|  | 9943, A->G | G->S | 9943, A->G | G->S | 9943, A->G | G->S | mutation 2 | TMP |
|  | 14672, G->T | R->I | 14672, G->T | R->I | 14672, G->T | R->I | mutation 5 | BP |
|  | 14900, T->C | V->A | 14900, T->C | V->A | 14900, T->C | V->A | mutation 4 | BP |
|  | 25472, G->A | silent | 25472, G->A | silent | 25472, G->A | silent | mutation 6 | DNA polymerase subunit |
|  |  |  |  |  |  |  |  |  |
| **P008>Lysc2** | 9072, A ->T | R->S | -- |  | -- |  | mutation 1 | TMP |
|  | 9080, C->A | T->K | -- |  | -- |  | mutation 3 | TMP |
|  | 9943, A->G | G->S | 9943, A->G | G->S | 9943, A->G | G->S | mutation 2 | TMP |
|  | 24857, G->A | silent | 24857, G->A | silent | 24857, G->A | silent | mutation 7 | DNA polymerase subunit |
|  |  |  | 14672, G->T | R->I | 14672, G->T | R->I | mutation 5 | BP |
|  |  |  | 14900, T->C | V->A | 14900, T->C | V->A | mutation 4 | BP |
|  |  |  |  |  |  |  |  |  |
| **P008>Lys1358** | 9072, A ->T | R->S | 9072, A ->T | R->S | 9072, A ->T | R->S | mutation 1 | TMP |
|  | 9943, A->G | G->S | 9943, A->G | G->S | 9943, A->G | G->S | mutation 2 | TMP |
|  | 14672, G->T | R->I | -- |  | -- |  | mutation 5 | BP |
|  | 14900, T->C | V->A | -- |  | 14900, T->C | V->A | mutation 4 | BP |
|  |  |  | 9080, C->A | T->K | 9080, C->A | T->K | mutation 3 | TMP |
|  |  |  | 24878, G->A | silent | 24878, G->A | silent | mutation 8 | DNA polymerase subunit |
|  |  |  |  |  |  |  |  |  |
| **P008>LysFL3B** | 9072, A ->T | R->S | -- |  | -- |  | mutation 1 | TMP |
|  | 9080, C->A | T->K | -- |  | -- |  | mutation 3 | TMP |
|  | 9943, A->G | G->S | 9943, A->G | G->S | 9943, A->G | G->S | mutation 2 | TMP |
|  |  |  | 14900, T->C | V->A | 14900, T->C | V->A | mutation 4 | BP |
|  |  |  |  |  |  |  |  |  |
| **P008>LysEFAS** | 9072, A ->T | R->S | 9072, A ->T | R->S | 9072, A ->T | R->S | mutation 1 | TMP |
|  | 9943, A->G | G->S | 9943, A->G | G->S | 9943, A->G | G->S | mutation 2 | TMP |
|  | 14840, C->T | A->V | 14840, C->T | A->V | 14840, C->T | A->V | mutation 9 | BP |
|  | 15065, C->T | A->V | 15065, C->T | A->V | 15065, C->T | A->V | mutation 10 | Holin |
|  | 25274, G->A | silent | 25274, G->A | silent | 25274, G->A | silent | mutation 11 | DNA polymerase subunit |
|  |  |  |  |  |  |  |  |  |
| **P008>LysLfeau** | 9943, A->G | G->S | 9943, A->G | G->S | -- |  | mutation 2 | TMP |
|  |  |  |  |  | 14840, C->T | A->V | mutation 12 | BP |
|  |  |  |  |  |  |  |  |  |

BP, Baseplate protein ; TMP, Tape-measure protein.
