# Supplementary material for: Phage endolysins are adapted to specific hosts and are evolutionarily dynamic
Source: PLoS Biol. 2022 Aug 1;20(8):e3001740. doi: 10.1371/journal.pbio.3001740 (PMC9371310; doi:10.1371/journal.pbio.3001740)
Supplement: S2 Table — (DOCX) [file pbio.3001740.s015.docx]

| **Bacterial strains** | | **Characteristics** | **Origin** | |  |  |  |  |
| --- | --- | --- | --- | --- | --- | --- | --- | --- |
| ***E. coli*** | |  |  | |  |  |  |  |
| BL21(DE3)pLysS | | F^-^ *ompT hsdSB* (r_B_ ^-^ m_B_^-^) *dcm*^+^*gal* (DE3) pLysS (Cam^r^ ) | Stratagene | |  |  |  |  |
| BL21/pLys1358^28a^ | | *E. coli* BL21 (DE3) transformed with pLys1358^28a^ | This study | |  |  |  |  |
| BL21/pLysc2^28a^ | | *E. coli* BL21 (DE3) transformed with pLysc2^28a^ | This study | |  |  |  |  |
| BL21/pLysP008^28a^ | | *E. coli* BL21 (DE3) transformed with LysP008^28a^ | This study | |  |  |  |  |
| BL21/pLysP335^20a^ | | *E. coli* BL21 (DE3) transformed with pLys1358^28a^ | This study | |  |  |  |  |
| BL21/pLysP335^28a^ | | *E. coli* BL21 (DE3) transformed with pLysP335^28a^ | This study | |  |  |  |  |
| BL21/pLysP335ΔTDM^20a^ | | *E. coli* BL21 (DE3) transformed with pLys1358^28a^ | This study | |  |  |  |  |
| ***L. lactis*** | |  |  | |  |  |  |  |
| IL1403 | | *Lactococcus lactis* laboratory strain | [1] | |  |  |  |  |
| IL6288 | | *Lactococcus lactis* IL1403 lacking all its prophages | [2] | | |  |  |  |
| SMQ-419 | | *Lactococcus lactis* QE | [3] | |  |  |  |  |
| SMQ-447 | | *Lactococcus lactis* RL34#25 | This study | |  |  |  |  |
| SMQ-385/HER1204 | | *Lactococcus lactis* ML8 | [4] | |  |  |  |  |
| SMQ-388/HER1205 | | *Lactococcus lactis* 582 | [4] | |  |  |  |  |
| SMQ-386/HER1248 | | *Lactococcus lactis* IE-16 | [5] | |  |  |  |  |
| SMQ-452 | | *Lactococcus lactis* RD565 | This study | |  |  |  |  |
| SMQ-378/HER1463 | | *Lactococcus lactis* F7/2 | [6] | |  |  |  |  |
| SMQ-1021 | | *Lactococcus lactis* H265 | [7] | |  |  |  |  |
| SMQ-382/HER1344 | | *Lactococcus lactis* 111 | [8] | | | | |  |
| SMQ-450 | | *Lactococcus lactis* RD563 | [8] | |  |  |  |  |
| SMQ-436 | | *Lactococcus lactis* PJ401 | [8] | |  |  |  |  |
| MG1363 | | *Lactococcus lactis* laboratory strain | [9] | |  |  |  |  |
| SMQ-754 | | *Lactococcus lactis* NP49-7 | [10] | |  |  |  |  |
| SMQ-384/HER1361 | | *Lactococcus lactis* C10 | [8] | |  |  |  |  |
| SMQ-1426 | | *Lactococcus lactis* DM31 | This study | |  |  |  |  |
| SMQ-1456 | | *L. lactis* IL1403 transformed with pL2Cas9-LysP335 and pΔLysP335::LysP008 | This study | |  |  |  |  |
| SMQ-1457 | | *L. lactis* IL1403 transformed with pL2Cas9-LysP335 and pΔLysP335::Lysc2 | This study | |  |  |  |  |
| SMQ-1458 | | *L. lactis* IL1403 transformed with pL2Cas9-LysP335 and pΔLysP335::Lys1358 | This study | |  |  |  |  |
| SMQ-1459 | | *L. lactis* IL1403 transformed with pL2Cas9-LysP008 and pΔLysP335::LysP335ΔLysM1-2 | This study | |  |  |  |  |
| SMQ-1460 | | *L. lactis* IL1403 transformed with pL2Cas9-LysP008 and pΔLysP008::LysP335 | This study | |  |  |  |  |
| SMQ-1461 | | *L. lactis* IL1403 transformed with pL2Cas9-LysP008 and pΔLysP008::Lysc2 | This study | |  |  |  |  |
| SMQ-1462 | | *L. lactis* IL1403 transformed with pL2Cas9-LysP008 and pΔLysP008::Lys1358 | This study | |  |  |  |  |
| SMQ-1463 | | *L. lactis* IL1403 transformed with pL2Cas9-LysP008 and pΔLysP008::Lys LysLfeSau | This study | |  |  |  |  |
| SMQ-1464 | | *L. lactis* IL1403 transformed with pL2Cas9-LysP008 and pΔLysP008::LysFL3B | This study | |  |  |  |  |
| SMQ-1465 | | *L. lactis* IL1403 transformed with pL2Cas9-LysP008 and pΔLysP008::LysEfaS | This study | |  |  |  |  |
| SMQ-1466 | | *L. lactis* IL1403 transformed with pL2Cas9-LysP008 and pΔLysP008::Lys481 | This study | |  |  |  |  |
|  |  | |  |  |  |  |  |  |
| **Plasmids** | | **Characteristics^a^** | **Origin** | | | |  |  |
| pET-28a | | Expression vector; Kan^r^ | Novagen | | | |  |  |
| pETG-20A | | Expression vector; Amp^r^ | EMBL | | | |  |  |
| pL2Cas9 | | pTRKL2::CRISPR-Cas9, 12.2 kb | [11] | | | |  |  |
| pL2Cas9-LysP008 | | pL2Cas9 with a spacer targeting the endolysin of phage P008 | This study | | | |  |  |
| pL2Cas9-LysP335 | | pL2Cas9 with a spacer targeting the endolysin of phage P335 | This study | | | |  |  |
| pLys1358^28a^ | | pET-28a carrying *Lys1358* | This study | | | |  |  |
| pLysc2^28a^ | | pET-28a carrying *Lysc2* | This study | | | |  |  |
| pLysP008^28a^ | | pET-28a carrying *LysP008* | This study | | | |  |  |
| pLysP335^20a^ | | pET-g20a carrying *LysP335* | This study | | | |  |  |
| pLysP335^28a^ | | pET-28a carrying *LysP335* | This study | | | |  |  |
| pLysP335ΔTDM^20a^ | | pET-g20a carrying *LysP335Δ* | This study | | | |  |  |
| pΔLysP008::Lys481 | | pNZ123 having the repair template for *LysP008* substitution with *Lys481* | This study | | | |  |  |
| pΔLysP008::Lys1358 | | pNZ123 having the repair template for *LysP008* substitution with *Lys1358* | This study | | | |  |  |
| pΔLysP008::Lysc2 | | pNZ123 having the repair template for *LysP008* substitution with *Lysc2* | This study | | | |  |  |
| pΔLysP008::LysEfaS | pNZ123 having the repair template for *LysP008* substitution with *LysEfaS* | | This study | | | |  |  |
| pΔLysP008::LysFL3B | pNZ123 having the repair template for *LysP008* substitution with *LysFL3B* | | This study | | | |  |  |
| pΔLysP008::LysLfeSau | pNZ123 having the repair template for *LysP008* substitution with *LysLfeSau* | | This study | | | |  |  |
| pΔLysP008::LysP335 | pNZ123 having the repair template for *LysP008* substitution with *LysP335* | | This study | | | |  |  |
| pΔLysP335::Lys1358 | pNZ123 having the repair template for *LysP335* substitution with *Lys1358* | | This study | | | |  |  |
| pΔLysP335::Lysc2 | pNZ123 having the repair template for *LysP335* substitution with *Lysc2* | | This study | | | |  |  |
| pΔLysP335::LysP008 | pNZ123 having the repair template for *LysP335* substitution with *LysP008* | | This study | | | |  |  |
| pΔLysP335::ΔLysM1-2 | pNZ123 having the repair template for the deletion of LysP335 LysM domains | | This study | | | |  |  |
| pNZ123 | High copy number vector, Cmr, 2.5 kb | | [12] | | | | | |

| **Oligonucleotides** | **Sequence 5’ → 3’** | **Origin** |  |
| --- | --- | --- | --- |
| Cas9_S.pyo_F6 | GTTCTTAGTGCATATAACAAACATAGAGAC | [11] | |
| crRNA_S.pyo_R | CCAAGTAGCGAAGCGAGC | [11] | |
| Gateway_lysP335_Fw | GGGGACAAGTTTGTACAAAAAAGCAGGCTTAGAAAACCTGTACTTCCAGGGTAAACGGCTCATCAAAAAATC | This study | |
| Gateway_lysP335_Rv | GGGGACCACTTTGTACAAGAAAGCTGGGTTTATTACTAGTAGTTGAGTGTCTG | This study | |
| Gateway_lysP335_delsig_Fw | GGGGACAAGTTTGTACAAAAAAGCAGGCTTAGAAAACCTGTACTTCCAGGGTgcTAGCGGCGATCAGGGGG | This study | |
| Gateway_lysP335_ delsig _Rv | GGGGACCACTTTGTACAAGAAAGCTGGGTTTATTACTAGTAGTTGAGTGTCTGGCCAG | This study | |
| p008_control_F | TGGCACTTTCCCACCTGC | This study | |
| p008_control_R | TGAAGCTGAAAAACGAGCCG | This study | |
| P335_control_F | ACTTCACAATCTAATACAATTGC | This study | |
| P335_control_R | GTTAGCTACCAACTGAGC | This study | |
| pL2Cas9_LysP008_2_5’ | AAACTACAGACACAAAACGCTACTATGTATCTAAG | This study | |
| pL2Cas9_LysP008_2_3’ | AAAACTTAGATACATAGTAGCGTTTTGTGTCTGTA | This study | |
| pL2Cas9_LysP335_5’ | AAACGTGATAATCTTTCATCAATTGCGAGCCGTTTG | This study | |
| pL2Cas9_LysP335_3’ | AAAACAAACGGCTCGCAATTGATGAAAGATTATCAC | This study | |
| pNZins_F | CGCTAAAACGTCTCAGAAAC | [11] | |
| pNZins_R | GTGATGGTTATCATGCAGGATTG | [11] | |
| T7 | TAATACGACTCACTATAGGG | Novagen | |
| T7 terminator | GCTAGTTATTGCTCAGCGG | Novagen | |
| ^a^ Abbreviations: Cam^r^, chloramphenicol resistance; Kan^r^, kanamycin resistance ^b^ | | |  |

**References**

1. Chopin A, Chopin MC, Moillo-Batt A, Langella P. Two plasmid-determined restriction and modification systems in *Streptococcus lactis*. Plasmid. 1984;11(3):260-3. Epub 1984/05/01. doi: 10.1016/0147-619x(84)90033-7. PubMed PMID: 6087394.

2. Aucouturier A, Chain F, Langella P, Bidnenko E. Characterization of a prophage-free derivative strain of *Lactococcus lactis* ssp. lactis IL1403 reveals the importance of prophages for phenotypic plasticity of the host. Frontiers in Microbiology. 2018;9:2032-. doi: 10.3389/fmicb.2018.02032. PubMed PMID: 30233519.

3. Deveau H, Van Calsteren MR, Moineau S. Effect of exopolysaccharides on phage-host interactions in *Lactococcus lactis*. Applied and Environmental Microbiology. 2002;68(9):4364-9. Epub 2002/08/30. doi: 10.1128/aem.68.9.4364-4369.2002. PubMed PMID: 12200288; PubMed Central PMCID: PMCPMC124071.

4. Jarvis AW. Differentiation of lactic streptococcal phages into phage species by DNA-DNA homology. Applied and Environmental Microbiology. 1984;47(2):343-9. Epub 1984/02/01. doi: 10.1128/aem.47.2.343-349.1984. PubMed PMID: 6324674; PubMed Central PMCID: PMCPMC239672.

5. Saxelin ML, Nurmiaho EL, Korhola MP, Sundman V. Partial characterization of a new C3-type capsule-dissolving phage of *Streptococcus cremoris*. Can J Microbiol. 1979;25(10):1182-7. Epub 1979/10/01. doi: 10.1139/m79-183. PubMed PMID: 119574.

6. Schäfer A, Geis A, Neve H, Teuber M. Bacteriophage receptors of *Lactococcus lactis* subsp. 'diacetylactis' F7/2 and *Lactococcus lactis* subsp. *cremoris* Wg2-1. FEMS Microbiology Letters. 1991;62(1):69-73. Epub 1991/02/01. doi: 10.1016/0378-1097(91)90257-b. PubMed PMID: 1903350.

7. Doré L, Pageau G, Bourque-Leblanc F, Dupuis M, Lessard-Hurtubise R, Lacasse G, et al. Complete genome sequences of 10 lactococcal *Skunavirus* phages isolated from cheddar cheese whey samples in Canada. Microbiology Resource Announcements. 2021;10(15):e00098-21. Epub 2021/04/17. doi: 10.1128/mra.00098-21. PubMed PMID: 33858920; PubMed Central PMCID: PMCPMC8050962.

8. Deveau H, Labrie SJ, Chopin M-C, Moineau S. Biodiversity and classification of lactococcal phages. Applied and Environmental Microbiology. 2006;72(6):4338-46. doi: 10.1128/AEM.02517-05. PubMed PMID: 16751549.

9. Gasson MJ. Plasmid complements of *Streptococcus lactis* NCDO 712 and other lactic streptococci after protoplast-induced curing. Journal of Bacteriology. 1983;154(1):1-9. Epub 1983/04/01. doi: 10.1128/jb.154.1.1-9.1983. PubMed PMID: 6403500; PubMed Central PMCID: PMCPMC217423.

10. Boucher I, Vadeboncoeur C, Moineau S. Characterization of genes involved in the metabolism of alpha-galactosides by *Lactococcus raffinolactis*. Applied and Environmental Microbiology. 2003;69(7):4049-56. Epub 2003/07/04. doi: 10.1128/aem.69.7.4049-4056.2003. PubMed PMID: 12839781; PubMed Central PMCID: PMCPMC165199.

11. Lemay ML, Tremblay DM, Moineau S. Genome engineering of virulent lactococcal phages using CRISPR-Cas9. ACS Synthetic Biology. 2017;6(7):1351-8. Epub 2017/03/23. doi: 10.1021/acssynbio.6b00388. PubMed PMID: 28324650.

12. De Vos WM. Gene cloning and expression in lactic streptococci. FEMS Microbiology Letters. 1987;46(3):281-95. doi: https://doi.org/10.1016/0378-1097(87)90113-3.
